# Effects of Quercetin on Metabolic Dysfunction‐Associated Steatotic Liver Disease: A Systematic Review and Meta‐Analysis

**DOI:** 10.1002/fsn3.71358

**Published:** 2025-12-15

**Authors:** Dachuan Jin, Shunqin Jin, Tao Zhou, Guoping Sheng, Peng Gao, Guangming Li

**Affiliations:** ^1^ Translational Medicine Research Center Affiliated Infectious Disease Hospital of Zhengzhou University (Henan Provincial Infectious Disease Hospital, Zhengzhou Sixth People's Hospital) Zhengzhou People's Republic of China; ^2^ Department of Radiology Hebei Medical University Shijiazhuang People's Republic of China; ^3^ Department of Geriatric Medicine, Key Lab of Cardiovascular Proteomics of Shandong University Qilu Hospital of Shandong University Jinan People's Republic of China; ^4^ Key Laboratory of Artificial Organs and Computational Medicine in Zhejiang Province, Shulan (Hangzhou) Hospital Affiliated to Shulan International Medical College Zhejiang Shuren University Hangzhou People's Republic of China; ^5^ Department of Liver Disease The Sixth People's Hospital of Zhengzhou Zhengzhou People's Republic of China

**Keywords:** liver function, MASLD, meta‐analysis, quercetin, systematic review

## Abstract

This systematic review and meta‐analysis aimed to evaluate the effects of quercetin (QE) on liver function, lipid profiles, kidney function, anthropometric measures, hematological markers, and inflammatory markers in patients with metabolic dysfunction‐associated steatotic liver disease (MASLD). PubMed, Cochrane Library, Web of Science, and Embase were searched to January 26, 2025, for randomized controlled trials (RCTs). Additional gray literature sources, trial registries, and preprint platforms were screened. Weighted mean differences (WMDs) with 95% confidence intervals were calculated. Heterogeneity was assessed using the *I* ^2^ statistic, and meta‐regression analyses explored the influence of quercetin dose, baseline BMI, and age. Seven RCTs (540 patients) were included. Quercetin significantly reduced liver enzymes (ALT, AST, GGT), direct bilirubin, and C‐reactive protein. Lipid profiles improved with reductions in total cholesterol, LDL, and triglycerides, and an increase in HDL. No significant effects were observed on kidney function, BMI, body fat, hematological markers, or TNF‐α. Meta‐regressions suggested dosage, baseline BMI, and age may contribute to heterogeneity. According to GRADE, the certainty of evidence ranged from very low to moderate. QE shows preliminary evidence of improving liver function, lipid profiles, and inflammation in MASLD patients. However, given the small number of RCTs and the generally limited certainty of evidence, these findings should be interpreted with caution. Further large‐scale, high‐quality trials are warranted to confirm its therapeutic role.

**PROSPERO Registration Code:** CRD42025639487

## Graphical abstract

This meta‐analysis evaluated the effects of quercetin on metabolic dysfunction‐associated steatotic liver disease (MASLD)‐related biomarkers. Quercetin significantly improved liver enzymes, lipid profiles, and reduced CRP and direct bilirubin levels, while showing no significant impact on kidney function, anthropometric parameters, or hematological markers. These findings support quercetin as a promising nutraceutical agent for MASLD management.

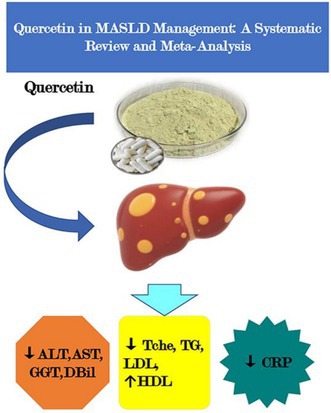

**Abbreviations**
95% CI: 95% confidence interval
AKP: alkaline phosphatase
ALT: alanine aminotransferase
AMPK: AMP‐activated protein kinase
AST: aspartate aminotransferase
ATF4: activating transcription factor 4
BMI: body mass index
BSEP: bile salt export pump
Cr: creatinine
CRP: C‐reactive protein
CYP7A1: cytochrome P450 7A1
DBil: direct bilirubin
EFSA: European Food Safety Authority
EGCG: Epigallocatechin Gallate
eIF2α: eukaryotic translation initiation factor 2 α
FBG: fasting blood glucose
FXR/NR1H4: farnesoid X receptor
GGT: gamma‐glutamyl transferase
GPX4: Glutathione Peroxidase 4
GRADE: the Grading of Recommendations Assessment, Development, and Evaluation
Hb: hemoglobin
HDL: high‐density lipoprotein
LDL: low‐density lipoprotein
LDLR: low‐density lipoprotein receptor
LFT: liver function test
MAFLD: metabolic dysfunction‐associated fatty liver disease
MASLD: metabolic dysfunction‐associated steatotic liver disease
mTOR: the mechanistic target of rapamycin
NAFLD: nonalcoholic fatty liver disease
NF‐κB: Nuclear Factor kappa B
NLRP3: NOD‐like receptor family, pyrin domain containing 3
Nrf2: Nuclear factor erythroid 2‐related factor 2
PCSK9: Proprotein Convertase Subtilisin/Kexin type 9
PERK: PKR‐like ER kinase
PPAR‐α: Peroxisome Proliferator‐Activated Receptor α
PRISMA: the Preferred Reporting Items for Systematic Reviews and Meta‐Analyses
PROSPERO: International prospective register of systematic reviews
QE: quercetin
RBC: red blood cells
RCT: randomized controlled trial
RoB2: Risk of Bias 2
SHP: small heterodimer partner
SIRT1: Sirtuin 1
SREBP‐1: Sterol Regulatory Element‐Binding Protein 1
SREBP‐2: Sterol Regulatory Element‐Binding Protein 2
TBil: total bilirubin
Tche: total cholesterol
TG: triglyceride
TNF‐α: tumor necrosis factor‐α
TP: total protein
UDCA: ursodeoxycholic acid
ULK: Unc‐51 like autophagy activating kinase
WBC: white blood cells
WMD: weighted mean difference
YY1: Yin Yang 1

## 1 Introduction

Metabolic dysfunction‐associated steatotic liver disease (MASLD) is a newly proposed term that replaces nonalcoholic fatty liver disease (NAFLD) and is defined through a modified Delphi process as the presence of hepatic steatosis with metabolic dysfunction in the absence of harmful alcohol consumption (Tacke et al. 2022; Rinella et al. 2023; Jin et al. 2022). It encompasses a spectrum of liver pathologies associated with metabolic dysfunction, including hepatic steatosis, steatohepatitis, fibrosis, cirrhosis, and hepatocellular carcinoma (EASL, EASD, EASO 2024; Jin, Jin, et al. 2023). Additionally, MASLD is closely linked to several extrahepatic diseases, such as hypertension, diabetes, and cardiovascular diseases (Chan et al. 2024). As the most common cause of chronic liver disease worldwide, it is estimated that approximately one‐third of the global population is affected by this condition, with incidence rates continuing to rise (Jin, Jin, et al. 2023; Procyk et al. 2024). Therefore, MASLD represents a significant global public health challenge. However, treatment options for MASLD remain limited, underscoring the urgent need for interventions that can halt or reverse disease progression and reduce associated morbidity and mortality (Procyk et al. 2024; Zeng et al. 2024; Jin et al. 2024).

Quercetin (QE), a naturally occurring flavonoid found in fruits and vegetables such as tomatoes, onions, and grapes, has emerged as a promising candidate for MASLD management (Markowska et al. 2024; Jin, Jin, Liu, et al. 2025). Chemically identified as 3,3′,4′,5,7‐ pentahydroxyflavone, QE is a well‐defined small‐molecule compound with a stable molecular structure (C_15_H_10_O_7_). It exhibits potent antioxidant and anti‐inflammatory properties, which may counteract the oxidative stress central to MASLD pathogenesis (Sotiropoulou et al. 2021; Yang et al. 2019; Cano‐Martínez et al. 2021; Kobori et al. 2020). Preclinical and clinical studies suggest that QE exerts hepatoprotective effects in both acute and chronic liver injury models, potentially improving MASLD by enhancing insulin sensitivity, reducing hepatic triglyceride accumulation, and attenuating liver damage (Yang et al. 2019; Sotiropoulou et al. 2021; Kim et al. 2015; Marcolin et al. 2013; Cao et al. 2023).

Despite these promising findings, current evidence on the therapeutic effects of QE remains fragmented and inconsistent (Jin et al. 2022; Hense et al. 2024; Stewart et al. 2009; Grosse et al. 2020). Some studies report significant improvements in liver enzymes and lipid profiles, while others show minimal or no benefit (Li et al. 2024; Hosseinikia et al. 2020; Kravchenko et al. 2023). This inconsistency may stem from differences in study design, treatment duration, patient characteristics, and outcome measures across trials. Moreover, the proposed mechanisms—such as AMPK activation and modulation of gut microbiota—vary substantially, further complicating the interpretation of QE's therapeutic potential (Nie et al. 2022; Mohammadhasani et al. 2024). Another critical source of inconsistency is the variation in the study population. Several studies have been conducted on healthy individuals or populations without a clearly defined MASLD diagnosis, thereby limiting their clinical relevance and generalizability. In contrast, findings from studies specifically involving patients with MASLD are more likely to reflect real‐world treatment responses, given the underlying metabolic impairment in this population (Stewart et al. 2009; Tabrizi et al. 2020; Huang et al. 2020). These inconsistencies and conflicting findings underscore the need for a systematic evaluation of clinical outcomes in patients with MASLD.

Previous systematic reviews on QE have primarily examined its effects in either preclinical models or human populations with general metabolic disorders, such as dyslipidemia or insulin resistance (Tabrizi et al. 2020; Huang et al. 2020; Sahebkar 2017; Guo et al. 2022). To date, no meta‐analysis has focused exclusively on patients diagnosed with MASLD based on the updated diagnostic criteria. Our study is, therefore, the first to quantitatively synthesize evidence from randomized controlled trials that specifically enrolled populations with clinically diagnosed MASLD. Given these inconsistencies and the promising therapeutic potential of QE, a comprehensive systematic review and meta‐analysis of clinical randomized controlled trials (RCTs) is warranted to synthesize the evidence and comprehensively evaluate its efficacy. This study aims to answer the following clinical question: In patients with MASLD, does quercetin supplementation, compared with placebo or standard care, improve liver enzymes, lipid profiles, renal function, anthropometric measures, hematological indicators, and inflammatory markers?

## 2 Materials and Methods

The protocol for this study has been registered in PROSPERO (Registration Code: CRD42025639487), and the study design follows the Preferred Reporting Items for Systematic Reviews and Meta‐Analyses (PRISMA) guideline (Liberati et al. 2009). Protocol details are available at https://www.crd.york.ac.uk/prospero/display_record.php?ID=CRD42025639487. A literature search was conducted in PubMed, Embase, Web of Science, and Cochrane Library for articles published from database inception to January 26, 2025, with no language restrictions. Based on the search strategies used in other published systematic reviews, our search strategy follows the example in PubMed, as shown in Table 1 (Lai et al. 2023; Zhou et al. 2024). To maximize sensitivity and avoid unnecessary exclusion of relevant trials, outcome‐related terms were not included in the search strategy. The search strategies for Embase, Cochrane Library, and Web of Science are provided in the Supporting Information as Tables S1–S3, respectively.

**TABLE 1:** Search strategy on PubMed.

#1	“quercetin” [MeSH Terms]
#2	“Quercetin” [Title/Abstract] OR “3 3 4 5 7 pentahydroxyflavone” [Title/Abstract] OR “Dikvertin” [Title/Abstract]
#3	#1 OR #2
#4	“non‐alcoholic fatty liver disease” [MeSH Terms]
#5	“metabolic associated fatty liver disease” [Title/Abstract] OR “MAFLD” [Title/Abstract] OR “metabolic dysfunction associated fatty liver disease” [Title/Abstract] OR “MASLD” [Title/Abstract] OR “NAFLD” [Title/Abstract] OR “non alcoholic fatty liver disease” [Title/Abstract] OR “non alcoholic fatty liver disease” [Title/Abstract] OR “fatty liver nonalcoholic” [Title/Abstract] OR (“fatty liver” [MeSH Terms] OR (“Fatty” [All Fields] AND “Liver” [All Fields]) OR “fatty liver” [All Fields] OR (“Fatty” [All Fields] AND “Livers” [All Fields]) OR “fatty livers” [All Fields] AND “Nonalcoholic” [Title/Abstract]) OR “liver nonalcoholic fatty” [Title/Abstract] OR ((“Liver” [MeSH Terms] OR “Liver” [All Fields] OR “Livers” [All Fields] OR “livers” [All Fields]) AND “nonalcoholic fatty” [Title/Abstract]) OR “nonalcoholic fatty liver” [Title/Abstract] OR “nonalcoholic fatty livers” [Title/Abstract] OR “NAFLD” [Title/Abstract] OR “nonalcoholic fatty liver disease” [Title/Abstract] OR “nonalcoholic steatohepatitis” [Title/Abstract] OR ((“fatty liver” [MeSH Terms] OR (“Fatty”[All Fields] AND “Liver” [All Fields]) OR “fatty liver” [All Fields]) AND “Nonalcoholic” [Title/Abstract]) OR “steatohepatitis nonalcoholic” [Title/Abstract] OR “metabolic associated steatohepatitis” [Title/Abstract] OR “NASH” [Title/Abstract] OR “steatosis of liver” [Title/Abstract] OR “liver steatosis” [Title/Abstract]
#6	#4 OR #5
#7	#3 AND #6

To minimize publication bias, we additionally searched major clinical trial registries (ClinicalTrials.gov, WHO ICTRP, ChiCTR, and EU Clinical Trials Register) and gray literature databases ProQuest and BASE. Selected preprint platforms (medRxiv, bioRxiv, SSRN) were also screened. We also performed citation searching (backward and forward) to identify additional potentially eligible studies.

### 2.1 Screening and Analysis

Two independent researchers performed an initial screening and data extraction of the literature based on the title and abstract, focusing on clinical studies with a randomized controlled design. Additionally, a manual search was performed to identify relevant articles and locate additional potentially relevant studies from the references. Any discrepancies or disagreements were resolved through group discussion.

### 2.2 Inclusion and Exclusion Criteria

Studies meeting the following criteria were considered eligible for inclusion: (1) Population: Patients diagnosed with NAFLD, MAFLD, or MASLD; (2) Intervention: Quercetin, regardless of the specific treatment regimen; (3) Control: Placebo or other comparable treatment; (4) Outcomes: Evaluation of therapeutic effects in six aspects—liver function, kidney function, anthropometric, metabolic, hematologic, and inflammatory indicators; (5) Study design: Clinical studies with a randomized controlled design. No language restrictions.

Studies meeting the following exclusion criteria were not included: non‐clinical studies, such as in vitro and animal studies, or studies without a control group. Reviews, book chapters, guidelines or consensuses, case reports, bioinformatics analyses, conference abstracts, comments, letters, retrospective studies, and study protocols. Studies that do not provide the outcomes of interest can be pooled for analysis with data from other studies, as well as those specifically focusing on children and adolescents under 18 years old.

### 2.3 Data Extraction and Quality Assessment

We developed a standardized data extraction form to collect the following information: the first author's surname, year of publication, study location, intervention details (including dose, frequency, and duration), sample size, age, and outcomes of interest. One was randomly selected for inclusion in studies with duplicate publications of the same data. This process was independently completed by two researchers (S.J. and G.S.). If any discrepancies arose, they were resolved through a group discussion. The same measurement unit was used for all the included data. If the original articles provided different measurement units, conversions were made through calculations to standardize them. Finally, all the data were confirmed after a collective discussion by the research team.

For the quality assessment of the included studies, two researchers (P.G. and G.L.) independently evaluated the risk of bias using the Cochrane Risk of Bias 2.0 (RoB2) tool, the current standard recommended by the Cochrane Collaboration. RoB2 assesses five domains of bias in randomized trials: (1) randomization process, (2) deviations from intended interventions, (3) missing outcome data, (4) measurement of the outcome, and (5) selection of the reported result. Each domain was judged as “low risk”, “some concerns”, or “high risk”, and an overall risk‐of‐bias judgment was assigned accordingly. Discrepancies between reviewers were resolved through group discussion. The RoB2 assessments for all included studies are presented in Figures S1 and S2.

### 2.4 Data Analysis

All analyses in this study were conducted using Stata 15.1 (StataCorp, College Station, TX). This meta‐analysis evaluated the effects of QE on the health of patients with MASLD across six aspects: liver function tests (LFT) (including ALT [alanine aminotransferase], AST [aspartate aminotransferase], GGT [gamma‐glutamyl transferase], AKP [alkaline phosphatase], TBil [total bilirubin], DBil [direct bilirubin], TP [total protein], and albumin), kidney function (urea and creatinine), anthropometric measures (BMI [body mass index] and body fat), metabolic indicators (Tche [total cholesterol], HDL [high‐density lipoprotein], LDL [low‐density lipoprotein], TG [triglyceride], and FBG [fasting blood glucose]), hematological aspects (WBC [white blood cells], RBC [red blood cells], and Hb [hemoglobin]), and inflammatory markers (CRP [C‐reactive protein] and TNF‐α [tumor necrosis factor‐α]). All outcomes were measured in consistent units after unit conversion. Therefore, we used Weighted Mean Difference (WMD) as the effect measure to facilitate clearer clinical interpretation, with a 95% confidence interval (95% CI). Statistical heterogeneity among studies was assessed using the Cochrane *Q* test and *I* ^2^ statistic. A fixed‐effects model was applied when *I* ^2^ < 50%, whereas a random‐effects model was used when *I* ^2^ ≥ 50%.

To explore potential sources of heterogeneity, meta‐regression analyses were performed using a random‐effects model. QE dosage (mg/day), baseline BMI, and mean age were selected as covariates based on prior evidence suggesting their relevance to treatment response. In addition, subgroup analyses were conducted by intervention duration, categorized as short‐term (< 8 weeks) and long‐term (≥ 8 weeks), to examine potential time‐dependent effects of QE on treatment outcomes. If the number of studies available for each indicator is fewer than 10, publication bias will not be assessed due to insufficient data. If the number of studies is 10 or greater, funnel plot analysis will be performed to further evaluate publication bias.

### 2.5 GRADE Assessment of Evidence Quality

The overall quality of evidence for all outcomes was assessed using the Grading of Recommendations Assessment, Development, and Evaluation (GRADE) approach, as outlined in the GRADE Working Group guidelines (Guyatt et al. 2008; Balshem et al. 2011). This framework considers five key domains—risk of bias, inconsistency, indirectness, imprecision, and publication bias—to rate the certainty of the evidence as high, moderate, low, or very low. The GRADEpro GDT software (McMaster University, 2023) was used to generate the Summary of Findings table. Judgments were based on the methodological quality of the included studies, the magnitude and precision of effect estimates, and the consistency of findings across studies. The assessment was conducted by two reviewers (S.J. & T.J.), and discrepancies were resolved by consultation with a third reviewer (G.L.).

## 3 Results

### 3.1 Study Characteristics

**FIGURE 1:**
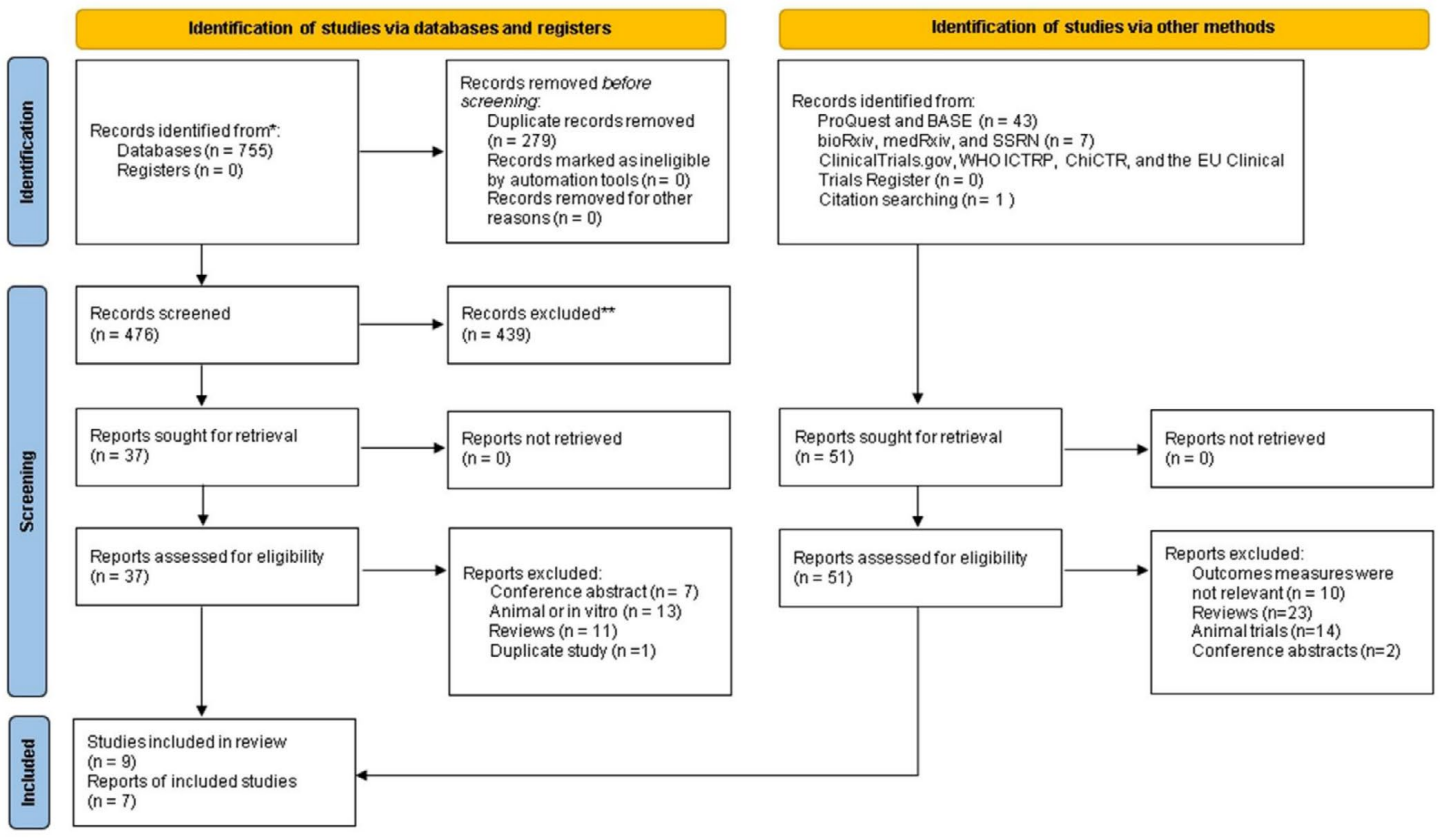
PRISMA flowchart of the literature selection process for this meta‐analysis. A total of 7 articles (reports) were included, representing 9 independent randomized controlled trials (studies). Five studies were identified via databases and registers, and two via other sources. One article contained data from three separate trials.

The PRISMA flowchart (Figure 1) summarized the complete literature retrieval and screening process. A total of 755 articles were obtained through a search of four databases, with an additional 51 articles identified through other sources (gray literature and manual screening of reference lists from included studies and related reviews).

After removing 279 duplicates, 476 articles remained. Subsequently, 439 articles were excluded following abstract screening. Full‐text reviews were conducted for the remaining 88 articles, including those identified through both database searches and manual reference screening, resulting in the exclusion of 81 articles. The reasons for exclusion include articles with only abstracts and no data, animal studies, reviews, and duplicate studies. Finally, seven articles were involved, encompassing nine treatment studies (Li et al. 2024; Hosseinikia et al. 2020; Kravchenko et al. 2023; Pasdar et al. 2020; Prysyazhnyuk and Voloshyn 2017; Teslenko et al. 2024; Khukhlina et al. 2020). A total of 540 participants were involved in the study. Among them, 274 were assigned to the QE group and 266 to the control group. All seven articles were published between 2017 and 2024. Geographically, one study was conducted in China (Li et al. 2024), two in Iran (Hosseinikia et al. 2020; Pasdar et al. 2020), and the others in Ukraine (Kravchenko et al. 2023; Prysyazhnyuk and Voloshyn 2017; Teslenko et al. 2024; Khukhlina et al. 2020). QE was administered orally in all studies, with a minimum dose of 40 mg three times daily and a maximum dose of 500 mg twice daily. Treatment duration varied across studies, with one Ukrainian study lasting 10 days (≈1.4 weeks), another 2 weeks, and the remaining seven studies 12 weeks (Table S4). Post‐treatment outcome values for all indicators are summarized in Table S5.

### 3.2 Risk of Bias Assessment

The risk of bias assessments using the Cochrane RoB2 tool are presented in Figures S1 and S2. Among the nine trials included in this review, six were judged to have “some concerns” due to potential issues in one or more of the following domains: deviations from intended interventions, measurement of the outcome, and the randomization process. The remaining three trials were assessed as having a low risk of bias, and no trials were rated as high risk.

### 3.3 Meta‐Analysis Results

#### 3.3.1 Impact on Liver Function

**FIGURE 2:**
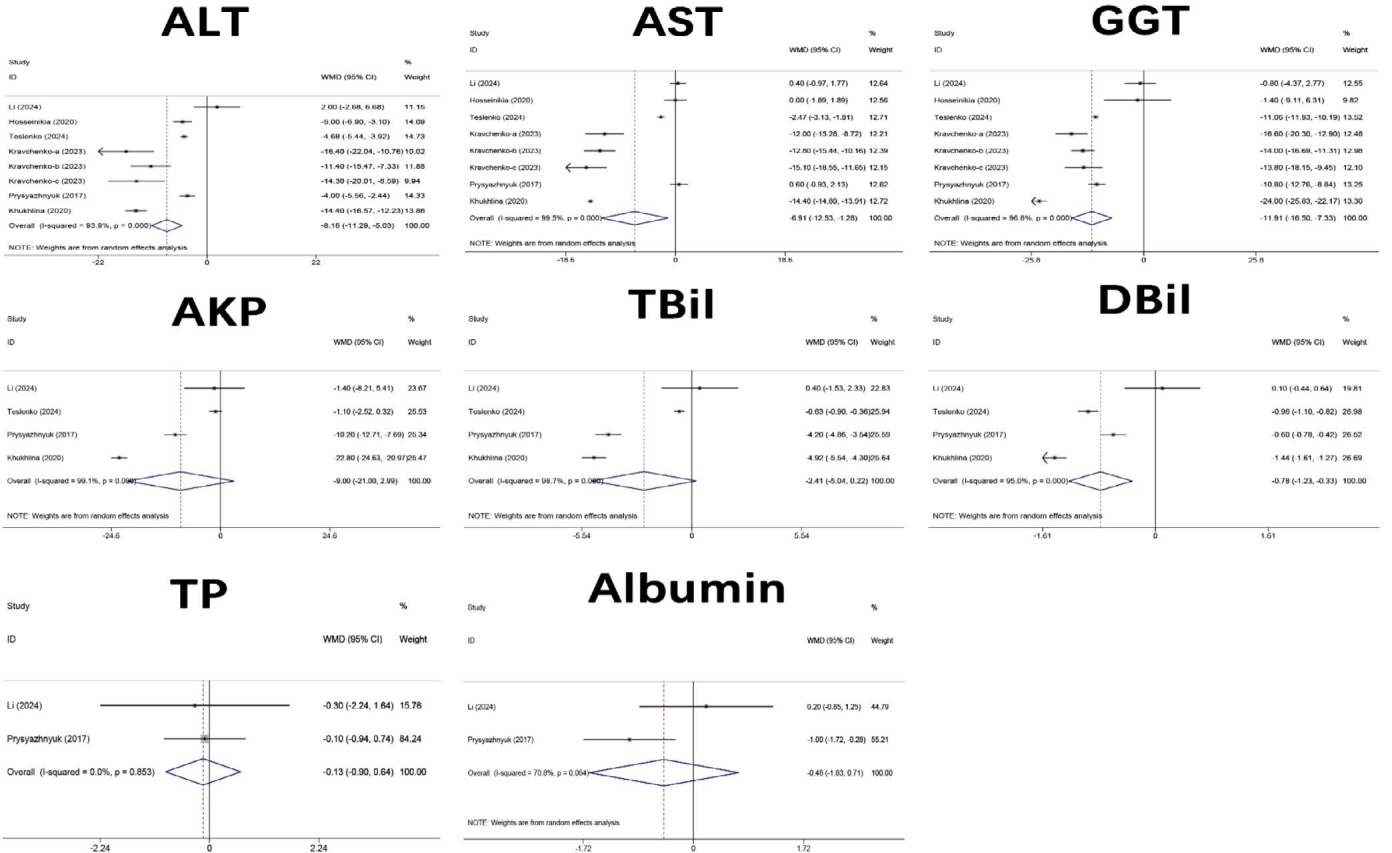
Forest plot analysis showing the effect of QE on liver function. AKP, alkaline phosphatase; ALT, Alanine aminotransferase; AST, Aspartate aminotransferase; DBil, direct bilirubin; GGT, gamma‐glutamyl transferase; TBil, total bilirubin; TP, total protein; QE, quercetin.

The reported indicators regarding the impact of QE on liver function included eight parameters: ALT, AST, GGT, AKP, TBil, DBil, TP, and albumin. There were six studies related to ALT (Li et al. 2024; Hosseinikia et al. 2020; Kravchenko et al. 2023; Prysyazhnyuk and Voloshyn 2017; Teslenko et al. 2024), AST (Li et al. 2024; Hosseinikia et al. 2020; Kravchenko et al. 2023; Prysyazhnyuk and Voloshyn 2017; Teslenko et al. 2024), and GGT (Li et al. 2024; Hosseinikia et al. 2020; Kravchenko et al. 2023; Prysyazhnyuk and Voloshyn 2017; Teslenko et al. 2024), three studies related to AKP (Li et al. 2024; Prysyazhnyuk and Voloshyn 2017; Teslenko et al. 2024), TBil (Li et al. 2024; Prysyazhnyuk and Voloshyn 2017; Teslenko et al. 2024), and DBil (Li et al. 2024; Prysyazhnyuk and Voloshyn 2017; Teslenko et al. 2024), and two studies related to TP (Li et al. 2024; Prysyazhnyuk and Voloshyn 2017) and albumin (Li et al. 2024; Prysyazhnyuk and Voloshyn 2017). Figure 2 summarizes the results of the pooled analysis indicating that supplementation with QE significantly reduced the levels of ALT (WMD: −8.16; 95% CI: −11.29 to −5.03), AST (WMD: −6.91; 95% CI: −12.53 to −1.28), GGT (WMD: −11.91; 95% CI: −16.50 to −7.33), and DBil (WMD: −0.78; 95% CI: −1.23 to −0.33). However, QE showed no statistically significant effect on the levels of AKP (WMD: −9.00; 95% CI: −21.00 to 2.99), TBil (WMD: −2.41; 95% CI: −5.04 to 0.22), TP (WMD: −0.13; 95% CI: −0.90 to 0.64), or albumin (WMD: −0.46; 95% CI: −1.63 to 0.71; Figure 2). Heterogeneity analysis indicated that the level of heterogeneity for TP (*I* ^2^ = 0.0%, *p* = 0.853) was low; therefore, a fixed‐effects model was used for the analysis. For ALT (*I* ^2^ = 93.95%, *p* < 0.001), AST (*I* ^2^ = 99.5%, *p* < 0.001), GGT (*I* ^2^ = 96.8%, *p* < 0.001), AKP (*I* ^2^ = 99.1%, *p* < 0.001), TBil (*I* ^2^ = 98.7%, *p* < 0.001), DBil (*I* ^2^ = 95.0%, *p* < 0.001), and albumin (*I* ^2^ = 70.8%, *p* = 0.064), since *I* ^2^ > 50%, a random‐effects model was employed for these analyses.

#### 3.3.2 Impact on Kidney Function

**FIGURE 3:**
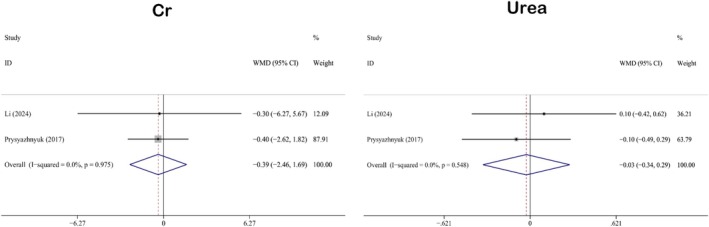
Forest plot analysis showing the effect of QE on kidney function. Cr, creatinine; QE, quercetin.

The indicators analyzed in this study regarding the impact on kidney function include creatinine (WMD: −0.39; 95% CI: −2.46 to 1.69) and urea (WMD: −0.03; 95% CI: −0.34 to 0.29). There were two studies associated with Cr and urea (Li et al. 2024; Prysyazhnyuk and Voloshyn 2017). No statistically significant differences were found between the experimental and control groups. Figure 3 presents the results of the forest plot analysis of kidney function indicators. Heterogeneity analyses demonstrated that both Cr (*I* ^2^ = 0.0%, *p* = 0.975) and urea (*I* ^2^ = 0.0%, *p* = 0.548) exhibited low levels of heterogeneity. Consequently, a fixed‐effects model was employed to analyze both indicators.

#### 3.3.3 Impact on Anthropometric Measurements

**FIGURE 4:**
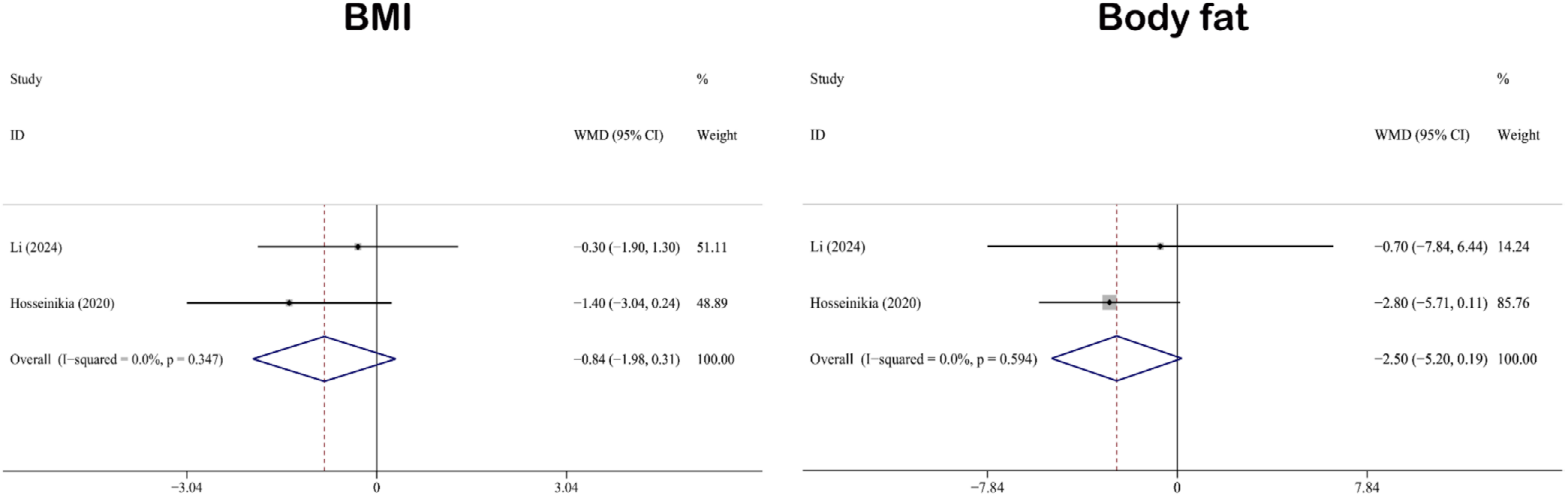
Forest plot analysis showing the effect of QE on anthropometric measurements. BMI, body mass index; QE, quercetin.

The anthropometric indicators included in this study were BMI (WMD: −0.84; 95% CI: −1.98 to 0.31) and body fat (WMD: −2.50; 95% CI: −5.20 to 0.19). Two studies were related to BMI and body fat (Li et al. 2024; Hosseinikia et al. 2020). No significant differences were observed between the experimental and control groups. Figure 4 summarizes the results of the forest plot analyses of anthropometric measures. Heterogeneity analyses showed that the heterogeneity levels of BMI (*I* ^2^ = 0.0%, *p* = 0.347) and body fat (*I* ^2^ = 0.0%, *p* = 0.594) were less than 50%, and the *p*‐values were greater than 0.05. Therefore, a fixed‐effects model was used for the analyses of both variables.

#### 3.3.4 Impact on Metabolic Indicators

**FIGURE 5:**
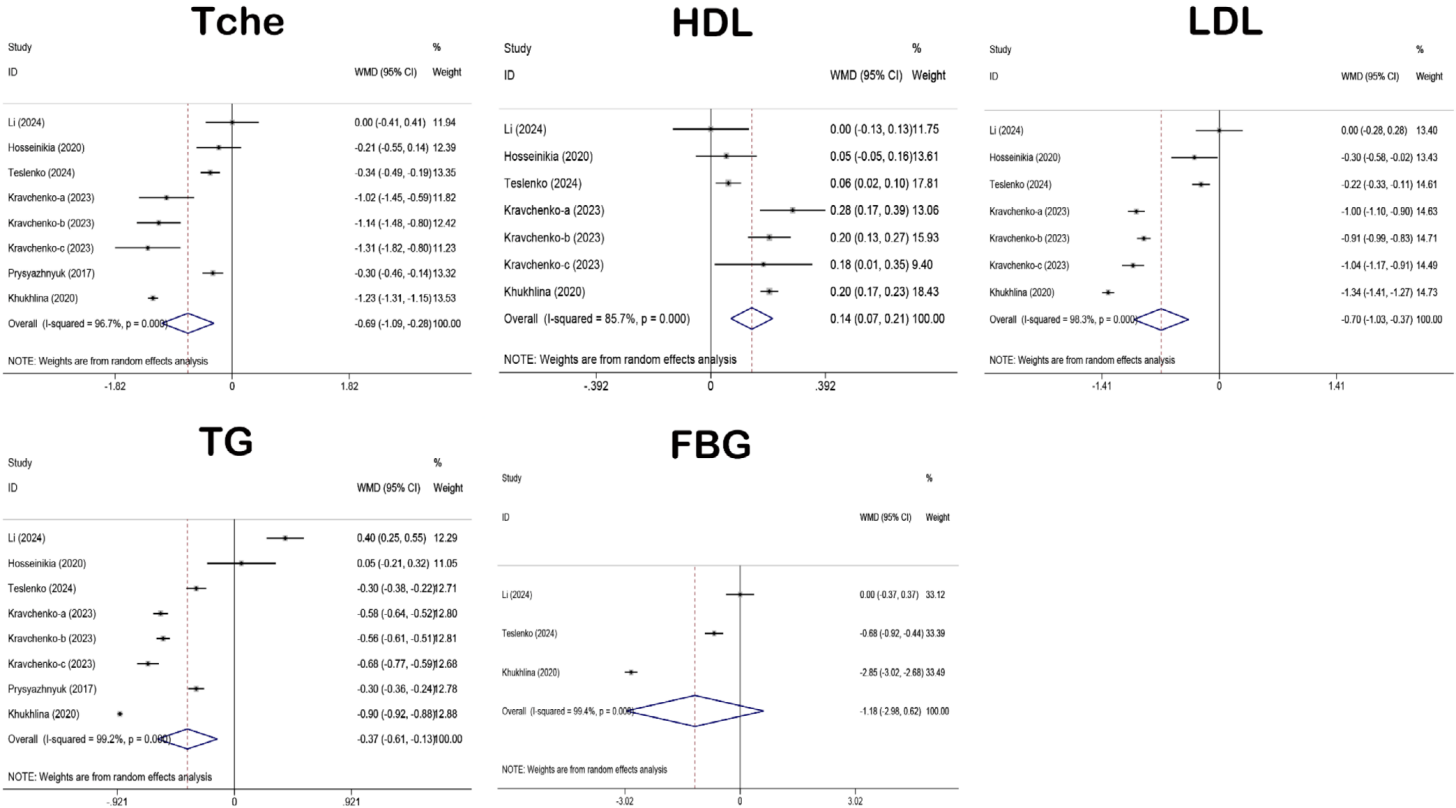
Forest plot analysis showing the effect of QE on metabolic indicators. FBG, fasting blood glucose; HDL, high‐density lipoprotein; LDL, low‐density lipoprotein; QE, quercetin; Tche, Total cholesterol; TG, triglycerides.

The metabolic indicators included in this study were Tche, HDL, LDL, TG, and FBG. There were six relevant studies from four articles each for Tche (Li et al. 2024; Hosseinikia et al. 2020; Kravchenko et al. 2023; Teslenko et al. 2024), HDL (Li et al. 2024; Hosseinikia et al. 2020; Kravchenko et al. 2023; Teslenko et al. 2024), LDL (Li et al. 2024; Hosseinikia et al. 2020; Kravchenko et al. 2023; Teslenko et al. 2024), and TG (Li et al. 2024; Hosseinikia et al. 2020; Kravchenko et al. 2023; Teslenko et al. 2024), whereas only two relevant studies were available for FBG (Li et al. 2024; Teslenko et al. 2024). Figure 5 illustrates the results of the forest plot analyses of the metabolic indicators. As can be gleaned from the figure, QE significantly improved Tche (WMD: −0.69; 95% CI: −1.09 to −0.28), HDL (WMD: 0.14; 95% CI: 0.07 to 0.21), LDL (WMD: −0.70; 95% CI: −1.03 to −0.37), and TG (WMD: −0.37; 95% CI: −0.61 to −0.13). However, no statistically significant effect was observed on FBG (WMD: −1.18; 95% CI: −2.98 to 0.62). Heterogeneity analyses indicated that *I* ^2^ value of Tche (*I* ^2^ = 96.7%, *p* < 0.001), HDL (*I* ^2^ = 85.7%, *p* < 0.001), LDL (*I* ^2^ = 98.3%, *p* < 0.001), TG (*I* ^2^ = 99.2%, *p* < 0.001), and FBG (*I* ^2^ = 99.4%, *p* < 0.001) were all greater than 50%, suggesting a high level of heterogeneity. Thus, a random‐effects model was employed to analyze all these metabolic indicators.

#### 3.3.5 Impact on Hematological Parameters

**FIGURE 6:**
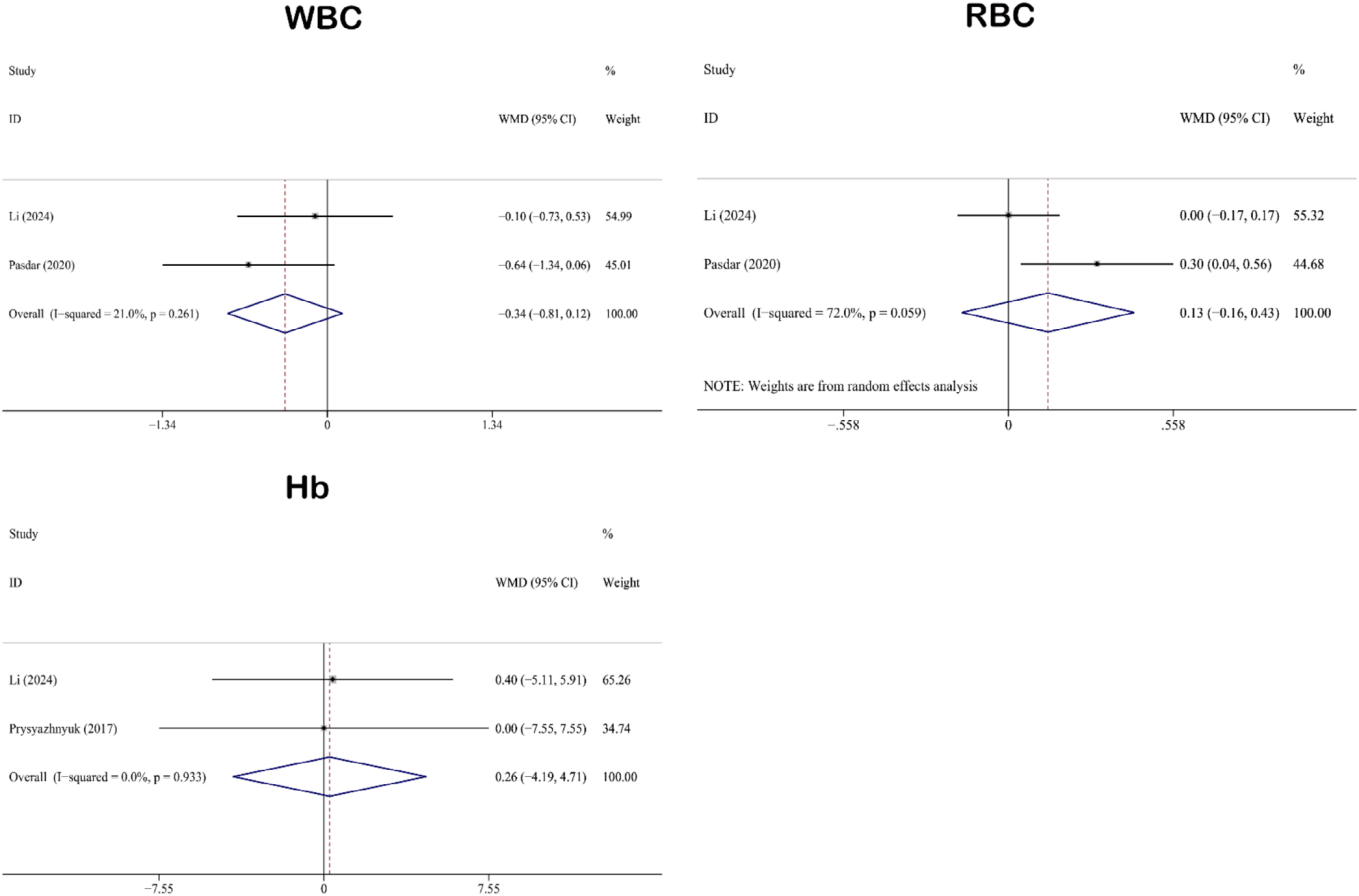
Forest plot analysis showing the effect of QE on hematological indicators. Hb, hemoglobin; QE, quercetin; RBC, red blood cells; WBC, white blood cells.

The hematological indicators reported in this study include WBC (WMD: −0.34; 95% CI: −0.81 to 0.12), RBC (WMD: 0.13; 95% CI: −0.16 to 0.43), and hemoglobin (WMD: 0.26; 95% CI: −4.19 to 4.71). There were two studies for each of WBC (Li et al. 2024; Pasdar et al. 2020), RBC (Li et al. 2024; Pasdar et al. 2020), and Hb (Li et al. 2024; Prysyazhnyuk and Voloshyn 2017). The results of the pooled analyses of the hematological indicators are shown in Figure 6. This figure reveals that no statistically significant effects of QE were observed on any of the aforementioned hematological parameters. Heterogeneity analyses demonstrated that the levels of heterogeneity for WBC (*I* ^2^ = 21.0%, *p* = 0.261) and Hb (*I* ^2^ = 0.0%, *p* = 0.933) were low. Therefore, a fixed‐effects model was used for the analyses. In contrast, the heterogeneity level of RBC (*I* ^2^ = 72.0%, *p* = 0.059) was high, so a random‐effects model was adopted for the analysis.

#### 3.3.6 Impact on Inflammation

**FIGURE 7:**
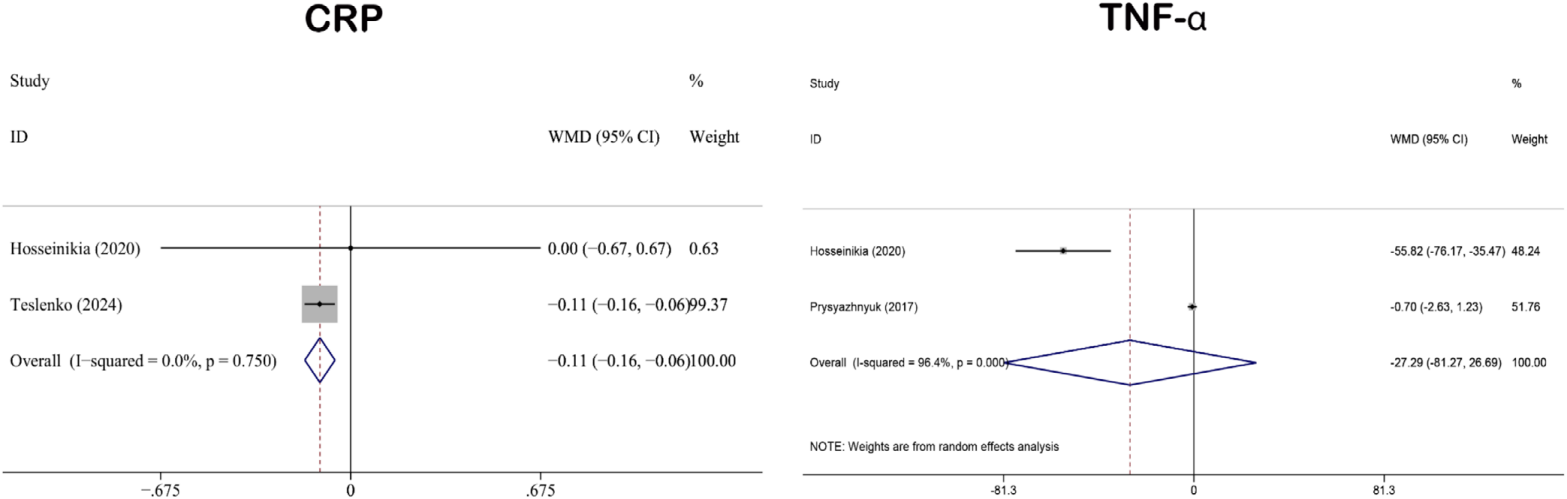
Forest plot analysis showing the effect of QE on inflammatory indicators. CRP, C‐reaction protein; QE, quercetin; TNF‐α, tumor necrosis factor‐α.

The inflammation‐related indicators included in this study were CRP (WMD: −0.11; 95% CI: −0.16 to −0.06) (Hosseinikia et al. 2020; Teslenko et al. 2024) and TNF‐α (WMD: −27.29; 95% CI: −81.27 to 26.69) (Hosseinikia et al. 2020; Prysyazhnyuk and Voloshyn 2017). There were two studies associated with both CRP and TNF‐α. The results of the pooled analyses are presented in Figure 7. As shown, QE significantly reduced CRP levels, whereas its effect on TNF‐α was not statistically significant. Heterogeneity analyses revealed that CRP had minimal heterogeneity (*I* ^2^ = 0.0%, *p* = 0.750), while TNF‐α showed substantial heterogeneity (*I* ^2^ = 96.4%, *p* < 0.001). Accordingly, a fixed‐effects model was used for CRP, and a random‐effects model was applied for TNF‐α to account for the observed between‐study variability.

### 3.4 Meta‐Regression and Subgroup Analyses

The results from Figures 2, 3, 4, 5, 6, 7 indicate that eight analyzed parameters (including TP, Cr, urea, BMI, body fat, WBC, Hb, and CRP) exhibit low heterogeneity, allowing the use of a fixed‐effects model. In contrast, the *I* ^2^ values for the remaining 14 parameters exceeded 50%, necessitating the application of a random‐effects model. We conducted meta‐regression analyses to assess the potential influence of QE dosage, baseline BMI, and mean age on treatment efficacy, following methodologies used in previous studies (Jin, Jin, et al. 2023; Zhu et al. 2022). As shown in Table S6, the results suggest that dosage, baseline BMI, and age may influence the therapeutic effects of QE. The presence of heterogeneity suggests that QE may exert greater efficacy in specific subgroups of MASLD, while its effects may be less pronounced in others. However, despite the observed heterogeneity in some parameters, most of the analyzed parameters showed a general trend of improvement, which further supports the reliability of our conclusions.

Previous animal studies have also found that the therapeutic effect of QE on inflammation is dose‐dependent, exhibiting a clear dose–response relationship (Mohammadi‐Sartang et al. 2017). Anti‐inflammatory effects are only observed at sufficiently high doses, while lower doses show no significant effect. Our findings confirm that the impact of QE on lipid metabolism and liver protection is also dose‐dependent. To achieve the desired therapeutic effects, it is essential to determine the effective therapeutic dose range.

In addition to dosage, the duration of intervention may also contribute to outcome variability. Subgroup analyses stratified by treatment duration (< 8 weeks vs. ≥ 8 weeks) were therefore conducted to examine potential time‐dependent effects. Among the 11 evaluated outcomes, a partially duration‐dependent pattern was observed. Significant reductions in ALT, AST, GGT, and Tche were mainly evident in the long‐duration subgroup (≥ 8 weeks), whereas short‐term interventions (< 8 weeks) produced comparable but mostly nonsignificant changes. Conversely, AKP, TBil, DBil, and FBG showed greater improvements in the short‐term subgroup, suggesting an early cholagogic or metabolic response. Both duration strata demonstrated favorable effects on LDL, HDL, and TG, although the magnitude of improvement was modest. Collectively, these findings indicate that longer intervention periods may be required to achieve consistent biochemical benefits, particularly for hepatic enzyme and lipid parameters (Figures S3 and S4).

### 3.5 Safety

Of the eight studies from seven articles incorporated into the analysis, three studies (Li et al. 2024; Hosseinikia et al. 2020; Pasdar et al. 2020) specifically investigated the side effects of QE, involving a total of 238 participants. The maximum dosage administered was 1000 mg orally per day for a continuous period of 12 weeks (Hosseinikia et al. 2020). Notably, no adverse effects were reported among any of the participants, indicating a favorable tolerance profile for QE. These findings are consistent with previous studies (Andres et al. 2018; Environmental Products of Design (EPOD) 2011). According to the European Food Safety Authority (EFSA) Guidelines, a daily dose of 1000 mg of QE continuously for 3 months is considered safe (Environmental Products of Design (EPOD) 2011). To date, there have been no reports on the maximum safe dosage of QE.

### 3.6 Certainty of Evidence Based on GRADE Assessment

The certainty of evidence was assessed for all reported outcomes using the GRADE approach. As shown in Table 2, the overall certainty ranged from very low to moderate across the 22 outcomes evaluated. For liver function indicators, the certainty was rated as very low for ALT, AST, GGT, AKP, TBil, and albumin due to serious concerns regarding the risk of bias, inconsistency, and imprecision. Moderate certainty was observed for DBil and TP. In the domain of kidney function, both creatinine and urea were graded as moderate certainty, while anthropometric measures such as BMI and body fat were rated as moderate and low, respectively, largely due to imprecision. Regarding metabolic outcomes, Tche, HDL, LDL, and FBG were assessed as moderate, while TG was downgraded to low due to inconsistency. Hematological indicators (WBC, RBC, Hb) and inflammatory markers (CRP, TNF‐α) were all rated as having moderate certainty, primarily limited by imprecision due to small sample sizes and wide confidence intervals. Overall, the moderate certainty of many outcomes indicates that further high‐quality studies are likely to influence the estimated effects. Detailed justifications and scoring across the five GRADE domains are presented in Table 2.

**TABLE 2:** Summary of GRADE assessment for all outcomes.

Quality assessment	Design	RoB	Inconsistency	Indirectness	Imprecision	Other considerations	No. of patients		Effect	Quality
No. of studies							QE	Control	WMD (95% CI)	
ALT (follow‐up 1.4–12 w)	Randomized trials	Serious	Serious	Not serious	Serious	None	235	227	−8.16 (−11.29, −5.03)	⨁◯◯◯
8										Very low
AST (follow‐up 1.4–12 w)	Randomized trials	Serious	Serious	Not serious	Serious	None	235	227	−6.91 (−12.53, −1.28)	⨁◯◯◯
8										Very low
GGT (follow‐up 1.4–12 w)	Randomized trials	Serious	Serious	Not serious	Serious	None	235	227	−11.91 (−16.50, −7.33)	⨁◯◯◯
8										Very low
AKP (follow‐up 1.4–12 w)	Randomized trials	Serious	Serious	Not serious	Serious	None	155	143	−9.00 (−21.00, 2.99)	⨁◯◯◯
4										Very low
TBil (follow‐up 1.4–12 w)	Randomized trials	Serious	Serious	Not serious	Serious	None	155	143	−2.41 (−5.04, 0.22)	⨁◯◯◯
4										Very low
DBil (follow‐up 1.4–12 w)	Randomized trials	Serious	Not serious	Not serious	Not serious	None	155	143	−0.78 (−1.23, −0.33)	⨁⨁⨁◯
4										Moderate
TP (follow‐up 2–12 w)	Randomized trials	Not serious	Not serious	Not serious	Serious	None	82	71	−0.13 (−0.90, 0.64)	⨁⨁⨁◯
2										Moderate
Albumin (follow‐up 2–12 w)	Randomized trials	Serious	Serious	Not serious	Serious	None	82	71	−0.46 (−1.63, 0.71)	⨁◯◯◯
2										Very low
Cr (follow‐up 2–12 w)	Randomized trials	Not serious	Not serious	Not serious	Serious	None	82	71	−0.39 (−2.46, 1.69)	⨁⨁⨁◯
2										Moderate
Urea (follow‐up 2–12 w)	Randomized trials	Not serious	Not serious	Not serious	Serious	None	82	71	−0.03 (−0.34, 0.29)	⨁⨁⨁◯
2										Moderate
BMI (follow‐up 2–12 w)	Randomized trials	Not serious	Not serious	Not serious	Serious	None	80	80	−0.84 (−1.98, 0.31)	⨁⨁⨁◯
2										Moderate
Body fat (follow‐up 12 w)	Randomized trials	Not serious	Not serious	Not serious	Serious	None	80	80	−2.50 (−5.20, 0.19)	⨁⨁◯◯
2										Low
Tche (follow‐up 1.4–12 w)	Randomized trials	Not serious	Not serious	Not serious	Serious	None	235	227	−0.69 (−1.09, −0.28)	⨁⨁⨁◯
8										Moderate
HDL (follow‐up 1.4–12 w)	Randomized trials	Not serious	Not serious	Not serious	Serious	None	194	197	0.14 (0.07, 0.21)	⨁⨁⨁◯
7										Moderate
LDL (follow‐up 1.4–12 w)	Randomized trials	Not serious	Not serious	Not serious	Serious	None	194	197	−0.70 (−1.03, −0.37)	⨁⨁⨁◯
7										Moderate
TG (follow‐up 1.4–12 w)	Randomized trials	Not serious	Serious	Not serious	Serious	None	235	227	−0.37 (−0.61, −0.13)	⨁⨁◯◯
8										Low
FBG (follow‐up 1.4–12 w)	Randomized trials	Not serious	Not serious	Not serious	Serious	None	114	113	−1.18 (−2.98, 0.62)	⨁⨁⨁◯
3										Moderate
WBC (follow‐up 12 w)	Randomized trials	Not serious	Not serious	Not serious	Serious	None	80	80	−0.34 (−0.81, 0.12)	⨁⨁⨁◯
2										Moderate
RBC (follow‐up 12 w)	Randomized trials	Not serious	Not serious	Not serious	Serious	None	80	80	0.13 (−0.16, 0.43)	⨁⨁⨁◯
2										Moderate
Hb (follow‐up 2–12 w)	Randomized trials	Not serious	Not serious	Not serious	Serious	None	80	80	0.26 (−4.19, 4.71)	⨁⨁⨁◯
2										Moderate
CRP (follow‐up 12 w)	Randomized trials	Not serious	Not serious	Not serious	Serious	None	80	83	−0.11 (−0.16, −0.06)	⨁⨁⨁◯
2										Moderate
TNF‐α (follow‐up 2–12 w)	Randomized trials	Not serious	Not serious	Not serious	Serious	None	80	69	−27.29 (−81.27, 26.69)	⨁⨁⨁◯
2										Moderate

## 4 Discussion

### 4.1 Principal Findings

To the best of our knowledge, although systematic reviews have been conducted on the therapeutic effects of QE in conditions such as obesity, blood pressure, and COVID‐19 (Serban et al. 2016; Cheema et al. 2023; Guo et al. 2019), this is the first comprehensive meta‐analysis to assess its efficacy in patients with MASLD specifically. Our pooled results from nine randomized controlled trials reported across seven articles involving 540 participants demonstrated that QE significantly improved liver function biomarkers (e.g., ALT, AST, and GGT), lipid profiles (e.g., Tche, LDL, HDL, and TG), and reduced serum levels of DBil and CRP, but had no significant effect on kidney function, anthropometric parameters, or hematological indices.

These findings highlight QE's potential as a hepatoprotective agent primarily through modulation of hepatic enzyme activity and bile acid homeostasis. Preclinical studies indicate that QE can modulate bile acid synthesis and transport, a mechanism that may underlie its effects on liver enzyme and bilirubin profiles in addition to its well‐recognized antioxidant properties (Son et al. 2019; Juárez‐Fernández et al. 2021; Petrov et al. 2019). Activation of the farnesoid X receptor (FXR/NR1H4) induces bile salt export pump (BSEP) protein expression and, via small heterodimer partner (SHP)‐mediated feedback, suppresses cytochrome P450 7A1 (CYP7A1), thereby enhancing bile acid efflux, facilitating bilirubin clearance, and alleviating intrahepatic cholestasis (Wang et al. 2024; Yang et al. 2023; Yuan et al. 2018). In addition, inhibition of the mechanistic target of rapamycin (mTOR)/Yin Yang 1 (YY1) signaling pathway further modulates CYP7A1 transcription, facilitating cholesterol‐to‐bile acid conversion and supporting detoxification (Yang et al. 2023; Yuan et al. 2018). Collectively, these mechanisms contribute to reductions in serum ALT, AST, GGT, and DBil levels observed in this meta‐analysis, reflecting decreased hepatocellular injury and improved bile excretory function. Importantly, these hepatocellular pathways are largely independent of QE's systemic anti‐inflammatory or antioxidant actions (Son et al. 2019; Petrov et al. 2019; Yang et al. 2023).

In terms of lipid regulation, QE significantly reduced Tche, LDL, and TG while increasing HDL. Dyslipidemia is central to MASLD pathophysiology (Yi et al. 2021), and several mechanisms may underpin QE's lipid‐lowering effects. QE has been reported to inhibit Proprotein Convertase Subtilisin/Kexin type 9 (PCSK9) secretion by activating Sterol Regulatory Element‐Binding Protein 2 (SREBP‐2) and suppressing Sortilin, thereby regulating cholesterol homeostasis via both low‐density lipoprotein receptor (LDLR)‐dependent and LDLR‐independent pathways. Elevated PCSK9 levels correlate with MASLD severity, hepatic inflammation, and steatosis (Han et al. 2024; Ruscica et al. 2016; Zhang et al. 2025). Moreover, QE modulates AMP‐activated protein kinase (AMPK)/Sterol Regulatory Element‐Binding Protein 1 (SREBP‐1), acetyl‐CoA carboxylase, and Peroxisome Proliferator‐Activated Receptor α (PPAR‐α) pathways, collectively suppressing hepatic lipogenesis and promoting fatty acid oxidation (Liu et al. 2024). Furthermore, QE glycosides, such as quercetin‐3‐rhamnoside, inhibit pancreatic lipase activity, thereby reducing dietary lipid absorption (Wang et al. 2023; Wu et al. 2021). QE has also been shown to modulate glycerophospholipid, sphingolipid, and linoleic acid metabolism, which may contribute to improved lipid homeostasis (Wang et al. 2023). The mechanisms underlying HDL elevation remain less clearly defined but may involve QE's antioxidative properties, which help reduce HDL oxidation and enhance reverse cholesterol transport (Li et al. 2004). Of note, while these effects are biologically plausible, some studies reported null or contradictory findings (Hense et al. 2024; Stewart et al. 2009), underscoring the importance of population selection, treatment duration, and formulation. Moreover, most mechanistic insights are derived from preclinical studies, and their clinical translation in MASLD populations remains preliminary.

Beyond bile acid regulation and lipid metabolism, QE exerts anti‐inflammatory and cytoprotective effects through multiple pathways. In preclinical rodent MASLD models and hepatocyte studies, QE inhibits Nuclear Factor kappa B (NF‐κB) signaling and NOD‐like receptor family, pyrin domain containing 3 (NLRP3) inflammasome activation, attenuates endoplasmic‐reticulum stress via the PKR‐like ER kinase (PERK)/eukaryotic translation initiation factor 2 α (eIF2α)/activating transcription factor 4 (ATF4) axis, activates Nuclear factor erythroid 2‐reated factor 2 (Nrf2)‐mediated antioxidant defenses, and engages the Sirtuin 1 (SIRT1)/AMPK/Unc‐51 like autophagy activating kinase (ULK) pathway to promote autophagy and mitochondrial quality control (Prysyazhnyuk and Voloshyn 2017; Andres et al. 2018; Cheema et al. 2023; Yuan et al. 2018; Park et al. 2023; Jin, Zhang, et al. 2023; Zhang et al. 2024, 2022; Wang et al. 2022; Li et al. 2021; Shu et al. 2024; Du et al. 2024). It also preserves Glutathione Peroxidase 4 (GPX4) activity and limits ferroptosis (Jiang et al. 2023; Deng et al. 2023; Peng et al. 2025). In humans, direct evidence for modulation of these signaling pathways is currently lacking; available clinical data show only downstream biomarker changes (e.g., modest CRP reduction in two trials), which do not establish causality with the above mechanisms. Consequently, while these pathways are biologically plausible and consistent across animal and cell models, their confirmation in human MASLD cohorts remains limited and warrants targeted translational studies.

Although encouraging, several outcome indicators did not reach statistical significance, including AKP, TBil, TP, albumin, creatinine, urea, FBG, BMI, body fat, WBC, RBC, Hb, and TNF‐α with QE intervention. The lack of statistically significant effects for these indicators may be attributed to several factors. First, the included trials used a wide range of QE doses (120 to 1000 mg/day), and previous studies indicate QE's biological effects are dose‐dependent (Mohammadi‐Sartang et al. 2017; Perez‐Vizcaino et al. 2009). This variability may obscure potential dose–response relationships. Moreover, inconsistent reporting of dosing rationale and bioavailability limits our ability to perform stratified analyses or determine threshold effects. Second, variability in participant characteristics (e.g., age, baseline weight) and intervention durations (10 days to 12 weeks) may have affected QE absorption and overall efficacy. These differences, together with the small number of studies, could partly explain the absence of statistically significant effects for some outcomes. It will be essential for future trials to adopt harmonized dosing protocols based on pharmacokinetic data, standardized durations, and clearly defined participant characteristics to improve comparability and strengthen the reliability of evidence. Such refinements would help validate QE's therapeutic potential in MASLD.

### 4.2 Clinical Implications

Beyond statistical significance, the magnitude of these changes warrants discussion in terms of clinical relevance. In our analysis, ALT was reduced by 8.16 U/L, representing nearly 20%–25% of the typical elevation observed in MASLD patients. Although no direct quantitative correlation exists between ALT reduction and histological improvement, such a decrease indicates a clinically meaningful attenuation of hepatocellular injury and a shift toward normalization, which is often considered a favorable prognostic indicator (Hoofnagle et al. 2013). LDL decreased by 0.7 mmol/L (~27 mg/dL); given that a 1 mmol/L reduction is associated with approximately 20%–25% fewer cardiovascular events in large‐scale trials, this change is likely to be clinically relevant, particularly since MASLD patients are at increased cardiometabolic risk (Baigent et al. 2010, 2005; Burger et al. 2024; Mihaylova et al. 2012). Similarly, TG decreased by ~0.37 mmol/L and HDL increased by ~0.14 mmol/L, reflecting improvements in lipid homeostasis and reverse cholesterol transport. Elevated TG is a core metabolic abnormality in MASLD, contributing to hepatic steatosis and systemic insulin resistance. Even modest reductions in TG can alleviate hepatic fat accumulation and reduce progression toward steatohepatitis (Sitthirach et al. 2022; Yang et al. 2024; Kakiyama et al. 2024; Tan et al. 2017; Frederico et al. 2011). Epidemiological studies suggest that each 0.1 mmol/L (~4 mg/dL) rise in HDL is associated with a 2%–3% reduction in cardiovascular risk (Pirillo et al. 2013); therefore, the ~0.14 mmol/L (~5.4 mg/dL) increase observed here may correspond to an approximately 3%–4% relative risk reduction. In MASLD, higher HDL levels are also linked to improved insulin sensitivity and reduced hepatic steatosis (Mandraffino et al. 2022; El Amrousy et al. 2022), implying that this moderate elevation could have both metabolic and hepatic clinical relevance, even if the causal role of HDL per se remains debated. CRP was modestly reduced by 0.11 mg/L across two trials. Given the minimal effect size and limited number of studies, its clinical significance is uncertain and should be interpreted cautiously.

### 4.3 Comparison With Other Antioxidants

Compared to established and investigational antioxidants for MASLD—such as vitamin E, resveratrol, and other polyphenols—QE exhibits a distinct profile characterized by broad mechanistic potential yet less comprehensive clinical validation. Vitamin E (α‐tocopherol) remains the most extensively supported antioxidant, demonstrating histological benefits in NASH; however, its long‐term use is limited by safety concerns, including an increased risk of hemorrhagic events and uncertain effects on fibrosis and cardiovascular outcomes (Sanyal et al. 2010; Miller et al. 2005; Klein et al. 2011; Gawrieh et al. 2021). Other polyphenols, such as resveratrol, curcumin, and Epigallocatechin Gallate (EGCG), show promising preclinical efficacy in modulating oxidative stress and inflammation, but their translation into consistent human outcomes has been hindered by poor bioavailability and underpowered clinical trials (Perumpail et al. 2018; Heebøll et al. 2014; Lukkunaprasit et al. 2023; Huang et al. 2024; Jin, Jin, Sheng, et al. 2025).

In this context, QE emerges with several distinctive advantages. Its multitargeted actions—encompassing antioxidant, anti‐inflammatory, and metabolic pathways—are comparable to those of other polyphenols. Nonetheless, QE offers additional practical benefits, including wide availability as a dietary flavonoid, a favorable long‐term safety profile, and low cost (Wiegand et al. 2010). Despite these advantages, its clinical development faces challenges similar to other polyphenolic agents, notably substantial pharmacokinetic variability and low oral bioavailability, which contribute to inconsistent trial results and a currently limited depth of clinical evidence.

Overall, while QE is a highly promising nutraceutical candidate for MASLD, its relative efficacy compared with agents such as vitamin E or resveratrol remains to be clearly defined. Future studies should prioritize head‐to‐head randomized trials to establish their comparative effectiveness, optimize dosing regimens to overcome bioavailability limitations, and explore potential synergistic combinations.

### 4.4 Strengths

This study has several distinct strengths. To our knowledge, this is the first meta‐analysis to exclusively evaluate the effects of QE in patients with MASLD based on updated diagnostic criteria, distinguishing it from previous reviews that included heterogeneous metabolic conditions or preclinical studies. It provides a timely and disease‐specific contribution to the evidence base for nutritional and phytochemical interventions in chronic liver disease. By focusing exclusively on RCTs involving clinically diagnosed MASLD patients, this study provides the first direct clinical‐level evidence of QE's therapeutic value in this specific population, addressing a critical gap in translational application.

By assessing six major domains ― liver enzymes, kidney function, anthropometric indices, hematological parameters, inflammatory markers, and lipid metabolism—our analysis provides a comprehensive evaluation of QE's therapeutic profile. Importantly, we imposed no language restrictions during the literature search, which minimizes the risk of regional publication bias and ensures a globally inclusive evidence base. All included studies were randomized controlled trials, strengthening the methodological rigor and internal validity of the pooled findings. Furthermore, meta‐regression analyses were conducted to investigate the potential moderating effects of dosage, BMI, and age, providing valuable insights for future trial design and personalized intervention strategies.

The observed reductions in liver enzymes, lipid parameters, and CRP suggest that QE may serve as a promising adjunctive intervention for MASLD patients. Given its favorable safety profile, low cost, and natural origin, QE supplementation could potentially be integrated into comprehensive MASLD management plans, particularly for patients seeking plant‐based or complementary therapeutic strategies. While not intended to replace established treatments, QE may offer metabolic and anti‐inflammatory benefits that complement lifestyle or pharmacological interventions. These results may inform early‐stage MASLD treatment strategies or integrative approaches alongside pharmacotherapy. However, before clinical adoption, larger and longer‐term trials are needed to define its optimal dose, duration, and target population.

### 4.5 Limitations

This study also has several limitations that warrant cautious interpretation. First, substantial heterogeneity was observed in several key hepatic enzyme outcomes (e.g., ALT, AST, and GGT; *I* ^2^ > 80%). Meta‐regression identified QE dosage, baseline BMI, and mean age as significant moderators, suggesting that treatment response varies with both dose intensity and patient characteristics. Higher dosages and lower baseline BMI were associated with greater reductions in transaminases and lipid parameters, supporting a potential dose–response relationship. These findings are consistent with preclinical evidence showing that QE's anti‐inflammatory and lipid‐modulating effects become more evident at sufficiently high doses (Sur et al. 2016; Kalantari et al. 2018). Treatment duration also contributed substantially to between‐study variance, as longer interventions (≥ 8 weeks) yielded greater improvements in hepatic and lipid parameters, such as ALT, AST, and Tche, whereas shorter interventions (< 8 weeks) showed stronger effects on biliary and glycemic markers. These time‐dependent patterns indicate that QE's hepatoprotective actions may follow distinct kinetic trajectories—with early biliary and glycemic responses preceding longer‐term enzymatic and lipid remodeling. Although such moderators partly explain the observed dispersion, residual heterogeneity persisted, likely due to differences in QE formulations, background therapies, and population characteristics.

In particular, one included study (Teslenko et al. 2024) administered QE in combination with ursodeoxycholic acid (UDCA), a bile acid–modulating and cytoprotective agent that shares overlapping mechanistic pathways with QE. Such co‐administration could have amplified reductions in liver enzymes and inflammatory markers, thereby inflating the pooled effects for ALT, AST, and CRP relative to QE monotherapy. Because concomitant pharmacotherapy was inconsistently reported in other trials, we were unable to perform a subgroup analysis to isolate this effect. To address this potential confounding, upcoming trials should explicitly document and, where possible, control for co‐interventions such as UDCA to clarify their contribution to between‐study variability. Interindividual variability in genetic polymorphisms or ethnic backgrounds may also influence quercetin metabolism and bioavailability, thereby contributing to differential treatment responses across populations. This clinical and methodological diversity inevitably reduces the certainty and generalizability of pooled estimates, leading to downgrading for inconsistency in the GRADE assessment.

Moreover, the substantial heterogeneity observed across studies further limits the certainty and generalizability of pooled estimates. In the context of high between‐study variance, pooled effects carry wider uncertainty and prediction intervals, reducing their transportability across populations and clinical settings. Methodological and clinical diversity—including variation in MASLD diagnostic definitions, QE formulations and dosing schedules, baseline risk profiles, and co‐interventions—complicates quantitative synthesis and contributes to downgrading for inconsistency in GRADE. Future trials should therefore adopt harmonized diagnostic criteria, standardized and transparently reported formulations and dosing regimens, consistent intervention durations, and predefined subgroup analyses (e.g., by dose and duration), alongside core outcome sets and protocol preregistration, to minimize heterogeneity and strengthen the robustness and interpretability of evidence.

Second, none of the studies assessed long‐term or structural outcomes, such as hepatic fibrosis, collagen deposition, or sustained redox homeostasis, which are critical for evaluating lasting hepatoprotective effects in chronic models. Most available data focused on short‐term biochemical changes rather than histopathological or functional recovery. The absence of such long‐term or histological endpoints limits the ability to determine whether QE's biochemical improvements translate into durable therapeutic benefits or structural liver protection. Future investigations should incorporate histological and mechanistic endpoints, such as fibrosis regression and redox balance, to clarify whether the biochemical responses observed are accompanied by true structural and functional liver recovery.

Third, most included studies had relatively short intervention durations, which may not be sufficient to capture the full spectrum of QE's metabolic and hepatoprotective effects. Such short‐term interventions could therefore underestimate its efficacy on slower‐evolving processes, such as lipid remodeling or oxidative stress adaptation. Longer‐term randomized trials are therefore needed to verify the persistence and time‐dependent evolution of QE's metabolic and hepatic benefits.

Fourth, the overall sample size remains relatively small, with only nine randomized controlled trials involving 540 participants. Although several outcomes, such as CRP, showed statistically significant improvements, others—including TNF‐α, hematological parameters, and FBG—did not reach significance, largely because each was assessed in only two to three small studies. The limited number of participants and studies for these endpoints substantially reduces the precision and robustness of pooled estimates. Under such conditions, non‐significant findings may reflect insufficient power rather than the absence of effect, while significant ones may be unstable because pooled estimates are highly sensitive to the inclusion or exclusion of single trials. Small sample sizes also increase the likelihood of type II error, meaning that potentially meaningful effects could remain undetected, and they hinder reliable evaluation of publication bias (Brown and Vavrek 2015; Mittendorf et al. 1995). From an evidence‐grading standpoint, such imprecision inevitably lowers the certainty of findings within the GRADE framework. Collectively, these constraints indicate that the current results—although encouraging—should be regarded as preliminary and require confirmation in larger, adequately powered, multicenter RCTs with harmonized designs.

Fifth, six of the nine included trials were rated as having “some concerns” in the RoB2 assessment, primarily due to deviations from intended interventions or outcome measurement, which may introduce performance and detection bias. Although no study was judged as high risk, these methodological limitations reduce the overall confidence in the pooled estimates. Consistently, the GRADE assessment rated most outcomes as moderate to low certainty, further underscoring the need for cautious interpretation of positive findings. Importantly, the prevalence of these methodological concerns indicates that even seemingly robust effect sizes should be interpreted as provisional signals rather than definitive evidence. This limitation represents a major caveat of our meta‐analysis and highlights the urgent need for rigorously designed, blinded, and adequately powered trials.

Sixth, for several outcomes, an *I* ^2^ value of 0% was observed, suggesting no detectable heterogeneity. However, this may reflect the small number of studies rather than true homogeneity (Tang et al. 2024; Cho et al. 2018). The limited power to detect between‐study variance may result in falsely low *I* ^2^ estimates, and underlying clinical or methodological differences may still exist but remain undetected (Grady et al. 2021; Zanen and Lammers 1995). Furthermore, while most outcomes showed favorable trends, some findings were inconsistent across studies. For example, the effect of QE on TNF‐α was highly heterogeneous, and the direction of effect varied. These inconsistencies may reflect differences in diagnostic criteria, adherence, background treatment, or unmeasured confounding.

Taken together, our findings suggest that QE shows preliminary clinical evidence of improving liver enzymes, lipid profiles, and systemic inflammation in MASLD patients. Nevertheless, these findings remain provisional given the limited evidence base. Future large‐scale, well‐designed RCTs with longer follow‐up are warranted to confirm these findings, define optimal dosing, and evaluate long‐term outcomes.

## 5 Conclusions

In summary, this meta‐analysis suggests that QE supplementation may exert beneficial effects on liver function and lipid metabolism in patients with MASLD, as evidenced by significant reductions in ALT, AST, GGT, DBil, Tche, LDL, and TG, along with an increase in HDL levels and a decrease in CRP levels. For other outcomes, such as kidney function, anthropometric measures, hematological markers, and TNF‐α, no statistically significant changes were observed, although a favorable trend was noted. Meta‐regression analysis further suggested that dosage, baseline BMI, and age may influence treatment response. However, given the small number of trials and the overall low‐to‐moderate certainty of evidence, these findings should be interpreted as preliminary. The current results mainly provide hypothesis‐generating evidence rather than definitive conclusions. Further large‐scale, rigorously designed randomized controlled trials with longer follow‐up are warranted to validate the findings, determine the optimal dosing strategy, elucidate the underlying mechanisms, and assess the long‐term clinical outcomes of QE in MASLD management.

## Author Contributions

**Dachuan Jin:** formal analysis (equal), writing – original draft (equal). **Shunqin Jin:** data curation (equal), investigation (equal), resources (equal). **Tao Zhou:** methodology (equal), software (equal). **Guoping Sheng:** data curation, investigation. **Peng Gao:** methodology, software. **Guangming Li:** project administration, validation.

## Funding

This study received support from the Zhengzhou Science and Technology Bureau through the 2024 Zhengzhou Municipal Science and Technology Innovation Guidance Program Project in the Medical and Health Field (Grant No.: 2024YLZDJH385).

## Ethics Statement

The authors have nothing to report.

## Consent

The authors have nothing to report.

## Conflicts of Interest

The authors declare no conflicts of interest.

## Supporting information


**Appendix S1:** fsn371358‐sup‐0001‐AppendixS1.docx.

## Data Availability

The article's supplementary data (not for publication) is available at http://10.6084/m9.figshare.28406843. Any additional data pertaining to this study can be procured from the corresponding authors upon reasonable request is made.
