# Supplementary material for: Effects of Quercetin on Metabolic Dysfunction‐Associated Steatotic Liver Disease: A Systematic Review and Meta‐Analysis
Source: Food Sci Nutr. 2025 Dec 15;13(12):e71358. doi: 10.1002/fsn3.71358 (PMC12703814; doi:10.1002/fsn3.71358)
Supplement: Supplementary file 1 — Appendix S1: fsn371358‐sup‐0001‐AppendixS1.docx. [file FSN3-13-e71358-s001.docx]

Supplementary Material

# Supplementary Tables

# Table S1 Search strategy on EmBase

| #1 | 'quercetin'/exp |
| --- | --- |
| #2 | '3, 3`, 4`, 5, 7 pentahydroxyflavone':ti,ab,kw OR 'ascorbic acid plus quercetin':ti,ab,kw OR 'flavin':ti,ab,kw OR 'hippuroflavin':ti,ab,kw OR 'meletin':ti,ab,kw OR 'meltin':ti,ab,kw OR 'quercetine':ti,ab,kw OR 'quercetol':ti,ab,kw OR 'quercetole':ti,ab,kw OR 'quercitin':ti,ab,kw OR 'quertine':ti,ab,kw OR 'sophoretin':ti,ab,kw OR 'quercetin':ti,ab,kw |
| #3 | #1 OR #2 |
| #4 | 'metabolic fatty liver'/exp OR 'nonalcoholic fatty liver'/exp |
| #5 | 'metabolic fatty liver':ti,ab,kw OR 'nonalcoholic fatty liver':ti,ab,kw OR 'dys-metabolism associated fatty liver disease':ti,ab,kw OR 'dysmetabolism associated fatty liver disease':ti,ab,kw OR 'dysmetabolism associated fld':ti,ab,kw OR mafld:ti,ab,kw OR 'metabolic associated fatty liver disease':ti,ab,kw OR 'metabolic associated fatty liver disease':ti,ab,kw OR 'metabolic dysfunction associated fatty liver disease':ti,ab,kw OR 'metabolic dysfunctionassociated fatty liver':ti,ab,kw OR 'metabolic fatty liver disease':ti,ab,kw OR 'metabolic fld':ti,ab,kw OR 'metabolism associated fatty liver disease':ti,ab,kw OR 'metabolic fatty liver':ti,ab,kw OR nafld:ti,ab,kw OR 'nonalcoholic fatty liver disease':ti,ab,kw OR 'non alcoholic fatty liver disease':ti,ab,kw OR 'non alcoholic hepato-steatosis':ti,ab,kw OR 'non alcoholic hepatosteatosis':ti,ab,kw OR 'non alcoholic liver steatosis':ti,ab,kw OR 'non alcoholic steatotic hepatopathy':ti,ab,kw OR 'non-alcoholic fatty liver':ti,ab,kw OR 'non-alcoholic fatty liver disease':ti,ab,kw OR 'non-alcoholic fld':ti,ab,kw OR 'non-alcoholic hepatic steatosis':ti,ab,kw OR 'nonalcoholic fatty liver disease':ti,ab,kw OR 'nonalcoholic fld':ti,ab,kw OR 'nonalcoholic hepatic steatosis':ti,ab,kw OR 'nonalcoholic hepatosteatosis':ti,ab,kw OR 'nonalcoholic liver steatosis':ti,ab,kw OR 'nonalcoholic fatty liver':ti,ab,kw |
| #6 | #4 OR #5 |
| #7 | #3 AND #6 |

# Table S2 Search strategy on Cochrane Library

| #1 | [Quercetin] explode all trees |
| --- | --- |
| #2 | (Quercetin):ti,ab,kw OR (3,3',4',5,7-Pentahydroxyflavone):ti,ab,kw OR (Dikvertin):ti,ab,kw |
| #3 | #1 OR #2 |
| #4 | [Non-alcoholic Fatty Liver Disease] explode all trees |
| #5 | (Non-alcoholic Fatty Liver Disease):ti,ab,kw OR (Non alcoholic Fatty Liver Disease):ti,ab,kw OR (Fatty Liver, Nonalcoholic):ti,ab,kw OR (Fatty Livers, Nonalcoholic):ti,ab,kw OR (Liver, Nonalcoholic Fatty):ti,ab,kw OR (Livers, Nonalcoholic Fatty):ti,ab,kw OR (Nonalcoholic Fatty Liver):ti,ab,kw OR (Nonalcoholic Fatty Livers):ti,ab,kw OR (NAFLD):ti,ab,kw OR (Nonalcoholic Fatty Liver Disease):ti,ab,kw OR (Nonalcoholic Steatohepatitis):ti,ab,kw OR (Nonalcoholic Steatohepatitides):ti,ab,kw OR (Steatohepatitides, Nonalcoholic):ti,ab,kw OR (Steatohepatitis, Nonalcoholic):ti,ab,kw OR (MAFLD):ti,ab,kw OR (metabolic associated fatty liver disease):ti,ab,kw OR (MASLD):ti,ab,kw OR (metabolic dysfunction-associated steatotic liver disease):ti,ab,kw OR (metabolic dysfunction-associated fatty liver disease):ti,ab,kw OR (NAFLD):ti,ab,kw OR (NASH):ti,ab,kw OR (MASH):ti,ab,kw OR (metabolic associated steatohepatitis):ti,ab,kw OR (steatosis of liver):ti,ab,kw OR (steatohepatitis nonalcoholic):ti,ab,kw OR (metabolic associated steatohepatitis):ti,ab,kw OR (liver steatosis):ti,ab,kw |
| #6 | #4 OR #5 |
| #7 | #3 AND #6 |

**Table S3 Search Strategy on Web of Science**

| #1 | TS=(Quercetin OR 3,3',4',5,7-Pentahydroxyflavone OR Dikvertin) |
| --- | --- |
| #2 | TS=(Non-alcoholic Fatty Liver Disease OR Non alcoholic Fatty Liver Disease OR Fatty Liver, Nonalcoholic OR Fatty Livers, Nonalcoholic OR Liver, Nonalcoholic Fatty OR Livers, Nonalcoholic Fatty OR Nonalcoholic Fatty Liver OR Nonalcoholic Fatty Livers OR NAFLD OR Nonalcoholic Fatty Liver Disease OR Nonalcoholic Steatohepatitis OR Nonalcoholic Steatohepatitides OR Steatohepatitides, Nonalcoholic OR Steatohepatitis, Nonalcoholic OR MAFLD OR metabolic associated fatty liver disease OR MASLD OR metabolic dysfunction-associated steatotic liver disease OR metabolic dysfunction-associated fatty liver disease OR NAFLD OR NASH OR MASH OR metabolic associated steatohepatitis OR steatosis of liver OR steatohepatitis nonalcoholic OR metabolic associated steatohepatitis OR liver steatosis) |
| #3 | #1 AND #2 |

| Author | Nation | Year | n | Age | Intervention | Time | Outcome of Interest |
| --- | --- | --- | --- | --- | --- | --- | --- |
| Li | China | 2024 | T:41 | 39.8±11.7 | T:QE 500mg/d | 12w | BMI, body fat, ALT, AST, GGT, AKP, TP, albumin, TBil, DBil, Cr, urea, FBG, Tche, HDL, LDL, WBC, RBC, Hb |
|  |  |  | C:41 | 39.8±11.7 | C:Placebo |  |  |
| Hosseinikia | Iran | 2020 | T:39 | 43.4±11.1 | T:QE 500mgx2/d | 12w | BMI, body fat, ALT, AST, GGT, Tche, HDL, LDL, TG, |
|  |  |  | C:39 | 45.9±9.2 | C:Placebo |  | CRP, TNF-α |
| Teslenko | Ukraine | 2024 | T:41 | 50.0±2.0 | T: UDCA+QE 500mg/d | 12w | ALT, AST, GGT, AKP, TBil, DBil, FBG, Tche, HDL, LDL, TG, CRP |
|  |  |  | C:44 | 52.5±2.0 | C: UDCA |  |  |
| Kravchenko-a | Ukraine | 2023 | T:11 | NA | T:QE 40mgx3/d | 12w | ALT, AST, GGT, Tche, HDL, LDL, TG |
|  |  |  | C:12 | NA | C:Placebo |  |  |
| Kravchenko-b | Ukraine | 2023 | T:22 | NA | T:QE 40mgx3/d | 12w | ALT, AST, GGT, Tche, HDL, LDL, TG |
|  |  |  | C:23 | NA | C:Placebo |  |  |
| Kravchenko-c | Ukraine | 2023 | T:8 | NA | T:QE 40mgx3/d | 12w | ALT, AST, GGT, Tche, HDL, LDL, TG |
|  |  |  | C:10 | NA | C:Placebo |  |  |
| Pasdar | Iran | 2020 | T:39 | 43.5±11.1 | T:QE 500mgx2/d | 12w | WBC,RBC,Hb |
|  |  |  | C:39 | 45.9±9.2 | Cl:Placebo |  |  |
| Khukhlina | Ukraine | 2020 | T:32 | NA | T:ST+QE 500mg/d | 1.4w | ALT, AST, GGT, AKP, TBil, DBil, Tche, HDL, LDL, TG, FBG |
|  |  |  | C:28 | NA | C:ST |  |  |
| Prysyazhnyuk | Ukraine | 2017 | T:41 | NA | T:QE 40mgx3/d | 2w | ALT, AST, GGT, AKP, TP, albumin, TBil, DBil, Cr, urea, Tche, TG,TNF-α |
|  |  |  | C:30 | NA | C:Placebo |  |  |

**Table S4 Characteristics of included studies**

Note: ALT: alanine aminotransferase; AST: aspartate aminotransferase; GGT, gamma-glutamyl transferase; AKP, alkaline phosphatase; TBil, total bilirubin; DBil, direct bilirubin; TP, total protein; QE, quercetin, Cr, creatinine; BMI, body mass index; Tche: total cholesterol; HDL, high-density lipoprotein; LDL, low-density lipoprotein; TG, triglycerides; FBG, fasting blood glucose; WBC, white blood cells; RBC, red blood cells; Hb, hemoglobin; CRP, C-reaction protein; TNF-α, tumor necrosis factor-alpha; T, treatment; C, control; NA, not available; UDCA, ursodeoxycholic acid; d, day; w, week; m, month; mg, miligram; ST, standard therapy; QE, quercetin.

**Table S5 Summary of Post-Treatment Outcomes (Mean ± SD)**

|  | Li2024 | | Hosseinikia2020 | | Teslenko2024 | | Kravchenko-a2023 | | Kravchenko-b2023 | | Kravchenko-c2023 | | Pasdar2020 | | Prysyazhnyuk2017 | | Khukhlina2020 | |
| --- | --- | --- | --- | --- | --- | --- | --- | --- | --- | --- | --- | --- | --- | --- | --- | --- | --- | --- |
|  | QE | Ctrl | QE | Ctrl | QE | Ctrl | QE | Ctrl | QE | Ctrl | QE | Ctrl | QE | Ctrl | QE | Ctrl | QE | Ctrl |
| ALT | 38.1±  10.58 | 36.1±  11.05 | 22±  4.75 | 27±  3.75 | 41.59±  1.728 | 46.27±  1.853 | 76.10±  6.70 | 92.50±  7.10 | 79.00±  7.20 | 90.40±  6.70 | 78.00±  6.40 | 92.3±  5.8 | NA | NA | 27.8±  3.30 | 31.8±  3.32 | 53.4  ±3.6 | 67.8  ±4.8 |
| AST | 23.8±  2.25 | 23.4±  3.875 | 35±  4.5 | 35±  4.0 | 38.61±  1.42 | 41.08±  1.698 | 66.40±  4.20 | 78.40±  3.80 | 69.80±  4.00 | 82.60±  5.00 | 70.80±  3.80 | 85.90±  3.60 | NA | NA | 30.1±  4.21 | 29.5±  2.33 | 37.8  ±0.6 | 52.2  ±1.2 |
| GGT | 34±  7.3 | 34.8±  9.1 | 27.7±  19.7 | 29.1±  14.7 | 49.14±  2.3 | 60.2±  1.728 | 41.4±  3.8 | 58±  5.2 | 42.4±  4.2 | 56.4±  5 | 41.8±  4.3 | 55.6±  5 | NA | NA | 30..7±  4.56 | 41.5±  3.86 | 40.8  ±4.2 | 64.8  ±3.0 |
| AKP | 70±  13.6 | 71.4±  17.6 | NA | NA | 81.71±  3.238 | 82.81±  3.46 | NA | NA | NA | NA | NA | NA | NA | NA | 78.3±  6.28 | 88.5±  4.49 | 104.4  ±1.2 | 127.2  ±4.8 |
| TBil | 13±  4.5 | 12.6±  4.4 | NA | NA | 9.83±  0.738 | 10.46±  0.488 | NA | NA | NA | NA | NA | NA | NA | NA | 9.5±  1.63 | 13.7±  1.2 | 24.11  ±1.24 | 29.03  ±1.19 |
| DBil | 3.7±  1.3 | 3.6±  1.2 | NA | NA | 3.17±  0.393 | 4.13±  0.27 | NA | NA | NA | NA | NA | NA | NA | NA | 2.8±  0.31 | 3.4±  0.44 | 5.34  ±0.29 | 6.78  ±0.37 |
| TP | 75.2±  3.6 | 75.5±  5.2 | NA | NA | NA | NA | NA | NA | NA | NA | NA | NA | NA | NA | 71.1±  1.94 | 71.2±  1.65 | NA | NA |
| A | 45.6±  2.1 | 45.4±  2.7 | NA | NA | NA | NA | NA | NA | NA | NA | NA | NA | NA | NA | 43.4±  1.32 | 44.4±  1.67 | NA | NA |
| Cr | 69.1±  13.9 | 69.4±  13.7 | NA | NA | NA | NA | NA | NA | NA | NA | NA | NA | NA | NA | 85.3±  5.81 | 85.7±  3.69 | NA | NA |
| Urea | 41±  5.3 | 5.2±  1.1 | NA | NA | NA | NA | NA | NA | NA | NA | NA | NA | NA | NA | 41±  5.4 | 5.5±  0.41 | NA | NA |
| BMI | 27.4±  3.6 | 27.7±  3.8 | 29.1±  3.1 | 30.5±  4.2 | NA | NA | NA | NA | NA | NA | NA | NA | NA | NA | NA | NA | NA | NA |
| Body fat | 76.7±  16.4 | 77.4±  16.6 | 32.1±  6.36 | 34.9±  6.75 | NA | NA | NA | NA | NA | NA | NA | NA | NA | NA | NA | NA | NA | NA |
| Tche | 5.2±  0.9 | 5.2±  1.0 | 4.95±  0.656 | 5.158±  0.885 | 4.02±  0.4 | 4.36±  0.3 | 6.46±  0.56 | 7.48±  0.48 | 6.50±  0.62 | 7.64±  0.54 | 6.55±  0.58 | 7.86±  0.50 | NA | NA | 4.8±  0.45 | 5.1±  0.23 | 5.27  ±0.13 | 6.50  ±0.17 |
| HDL | 1.3±  0.3 | 1.3±  0.3 | 1.139±  0.253 | 1.09±  0.22 | 2.03±  0.105 | 1.97±  0.1 | 1.96±  0.15 | 1.68±  0.12 | 1.92±  0.12 | 1.72±  0.13 | 1.94±  0.2 | 1.76±  0.15 | NA | NA | NA | NA | 1.35  ±0.07 | 1.15  ±0.05 |
| LDL | 2.6±  0.6 | 2.6±  0.7 | 2.53±  0.607 | 2.83±  0.651 | 2.98±  0.29 | 3.2±  0.203 | 3.1±  0.13 | 4.1±  0.12 | 3.19±  0.15 | 4.1±  0.13 | 3.24±  0.14 | 4.28±  0.15 | NA | NA | NA | NA | 2.59  ±0.15 | 3.93  ±0.14 |
| TG | 2.3±  0.35 | 1.9±  0.325 | 1.736±  0.462 | 1.681±  0.724 | 1.63±  0.233 | 1.93±  0.113 | 1.00±  0.06 | 1.58±  0.08 | 1.08±  0.08 | 1.64±  0.10 | 1.12±  0.10 | 1.80±  0.08 | NA | NA | 1.5±  0.12 | 1.8±  0.14 | 1.54  ±0.03 | 2.44  ±0.05 |
| FBG | 5.7±  0.8 | 5.7±  0.9 | NA | NA | 4.85±  0.65 | 5.53±  0.43 | NA | NA | NA | NA | NA | NA | NA | NA | NA | NA | 5.7  5±0.19 | 8.6  ±0.41 |
| WBC | 6±  1.3 | 6.1±  1.6 | NA | NA | NA | NA | NA | NA | NA | NA | NA | NA | 6.1±  1.64 | 6.74±  1.5 | NA | NA | NA | NA |
| RBC | 5±  0.4 | 5±  0.4 | NA | NA | NA | NA | NA | NA | NA | NA | NA | NA | 5.22±  0.53 | 4.92±  0.63 | NA | NA | NA | NA |
| Hb | 150±  13.4 | 149.6±  12 | NA | NA | NA | NA | NA | NA | NA | NA | NA | NA | NA | NA | 140±  13.2 | 140±  20.1 | NA | NA |
| CRP | NA | NA | 1.53±  1.35 | 1.532±  1.673 | 1.65±  0.12 | 1.76±  0.13 | NA | NA | NA | NA | NA | NA | NA | NA | NA | NA | NA | NA |
| TNF-α | NA | NA | 97.7±  32.9 | 153.56  ±55.85 | NA | NA | NA | NA | NA | NA | NA | NA | NA | NA | 24.1  ±4.32 | 24.8  ±3.94 | NA | NA |

Note: ALT: alanine aminotransferase; AST: aspartate aminotransferase; GGT, gamma-glutamyl transferase; AKP, alkaline phosphatase; TBil, total bilirubin; DBil, direct bilirubin; TP, total protein; A, albumin; QE, quercetin; Ctrl, control; Cr, creatinine; BMI, body mass index; Tche: total cholesterol; HDL, high-density lipoprotein; LDL, low-density lipoprotein; TG, triglycerides; FBG, fasting blood glucose; WBC, white blood cells; RBC, red blood cells; Hb, hemoglobin; CRP, C-reaction protein; TNF-α, tumor necrosis factor-alpha; T, treatment; C, control; NA, not available; SD, standard deviation.

**Table S6 Meta-regression analysis of QE effects in patients with MASLD**

| Group | Indicator | Covariates | Coef | SE | z | p | 95%CI |
| --- | --- | --- | --- | --- | --- | --- | --- |
| LFT | ALT | dose | -1.87398 | 0.615559 | -3.04 | 0.0020 | -3.080449-0.6675023 |
|  |  | BMI | 16.72455 | 2.335426 | 7.16 | 0.0000 | 12.1472，21.3019 |
|  |  | age | 9.866915 | 1.426013 | 6.92 | 0.0000 | 7.071982，12.66185 |
|  | AST | dose | -2.52166 | 0.891215 | -2.83 | 0.0050 | -4.268404，-0.7749047 |
|  |  | BMI | 10.83167 | 3.852829 | 2.81 | 0.0050 | 3.280259,18.38307 |
|  |  | age | 6.411131 | 2.707955 | 2.37 | 0.0180 | 1.103637,11.71863 |
|  | GGT | dose | -3.54099 | 1.058649 | -3.34 | 0.0010 | -5.615904,-1.466075 |
|  |  | BMI | 34.27004 | 15.27787 | 2.24 | 0.0250 | 4.325955,64.21412 |
|  |  | age | -0.48464 | 0.231104 | -2.10 | 0.0360 | -0.9375991,-0.0316895 |
|  | AKP | dose | -2.33129 | 0.379471 | -6.14 | 0.0000 | -3.075044,-1.587545 |
| Metabolism | Tche | dose | -1.75032 | 0.418294 | -4.18 | 0.0000 | -2.570164,-0.930482 |
|  |  | BMI | 5.850794 | 2.024081 | 2.89 | 0.0040 | 1.883667,9.81792 |
|  |  | age | 3.436763 | 1.254684 | 2.74 | 0.0060 | 0.9776284,5.895899 |
|  | HDL | dose | 1.520585 | 0.400876 | 3.79 | 0.0000 | 0.7348833,2.306287 |
|  |  | BMI | -3.47151 | 1.987753 | -1.75 | 0.0810 | -7.367437,0.424412 |
|  |  | age | -2.04393 | 1.234594 | -1.66 | 0.0980 | -4.463685,0.3758323 |
|  | LDL | dose | -7.10098 | 1.59906 | -4.44 | 0.0000 | -10.23508,-3.966885 |
|  |  | BMI | 5.084194 | 2.015207 | 2.52 | 0.0120 | 1.134461,9.033927 |
|  |  | age | 2.997401 | 1.249754 | 2.40 | 0.0160 | 0.5479268,5.446874 |
|  | TG | dose | -6.17876 | 1.602864 | -3.85 | 0.0000 | -9.32031,-3.0372 |
|  |  | BMI | 18.02732 | 2.208571 | 8.16 | 0.0000 | 13.6986,22.35604 |
|  |  | age | 11.09797 | 1.368597 | 8.11 | 0.0000 | 8.415569,13.78037 |

Note: ALT: alanine aminotransferase; AST: aspartate aminotransferase; GGT, gamma-glutamyl transferase; AKP, alkaline phosphatase; Tche: total cholesterol; HDL, high-density lipoprotein; LDL, low-density lipoprotein; TG, triglycerides; LFT: liver function test; MASLD: metabolic dysfunctiono-associated steatotic fatty liver disease; QE, quercetin.

#
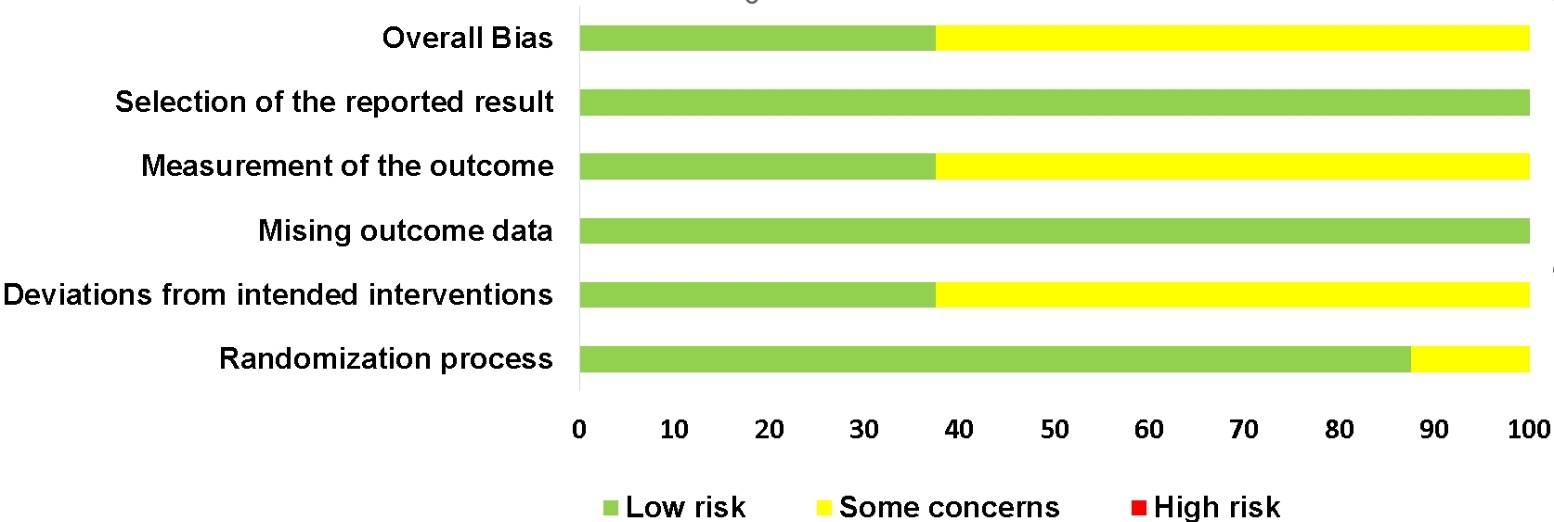
Supplementary Figures

**Supplementary Figure S1** Risk of Bias graph assessed with RoB2: review authors’ judgments about each risk of bias item presented as percentages across all included studies in this tria


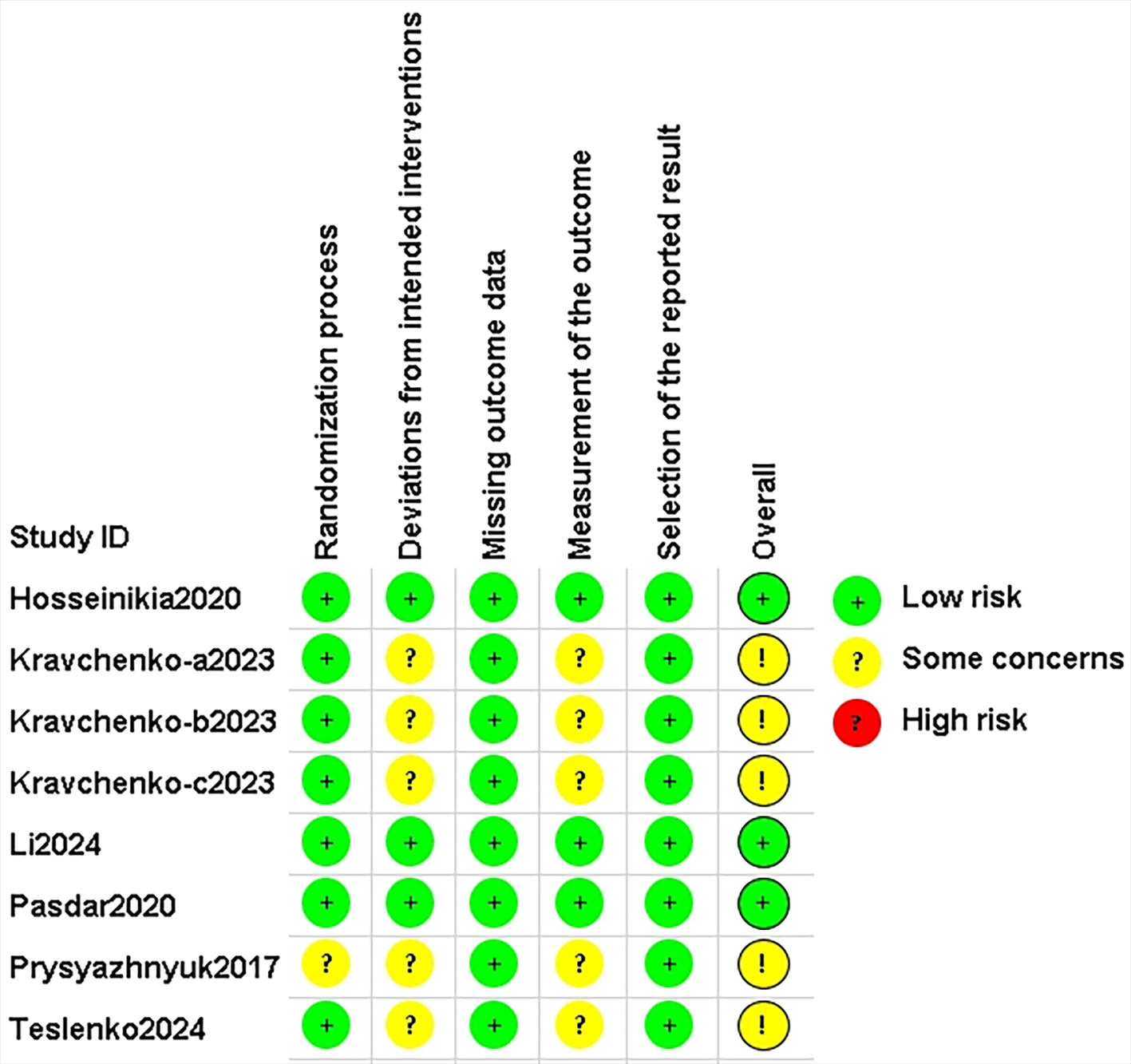


**Supplementary Figure S2** Risk of bias summary assessed with RoB2: review authors judgments about each risk of bias item for each included study in this trial


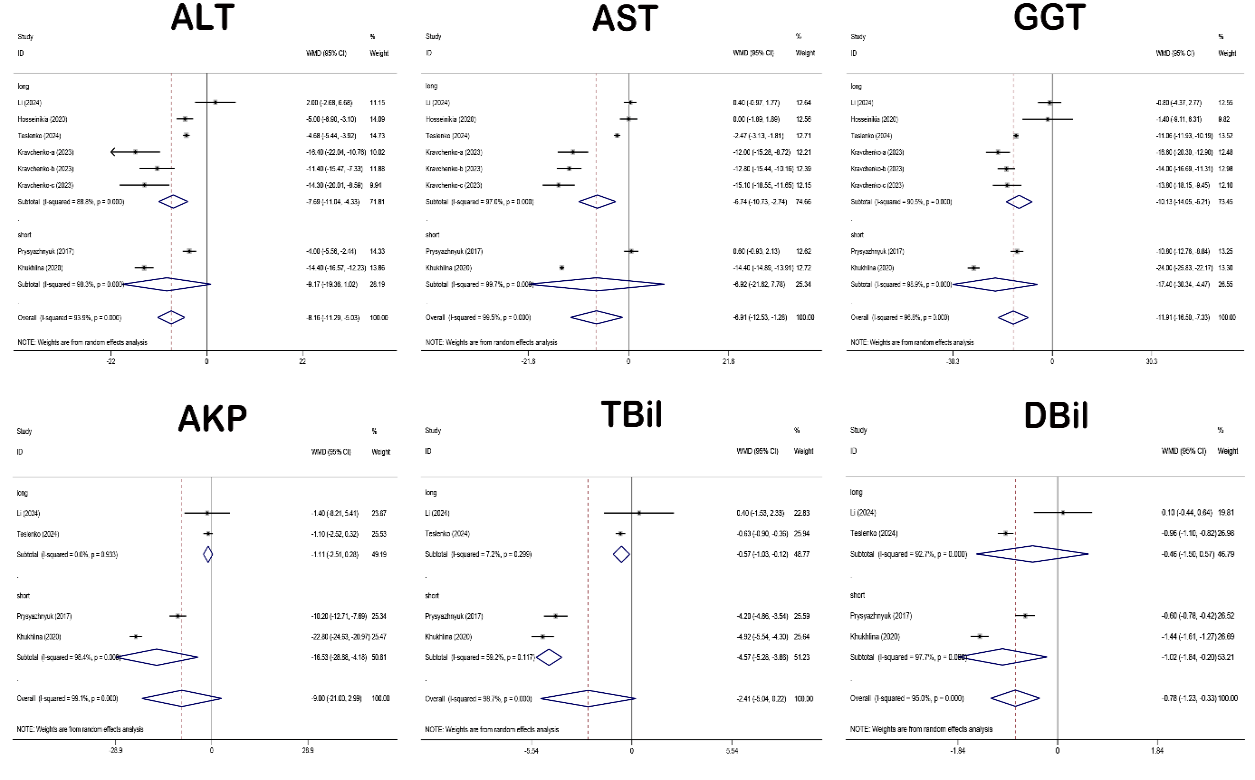


**Supplementary Figure S3** Forest plots of subgroup analyses by treatment duration on LFT. ALT: alanine aminotransferase; AST: aspartate aminotransferase; GGT: gamma-glutamyl transferase; AKP: alkaline phosphatase; Tbil: total bilirubin; DBiL, direct bilirubin.


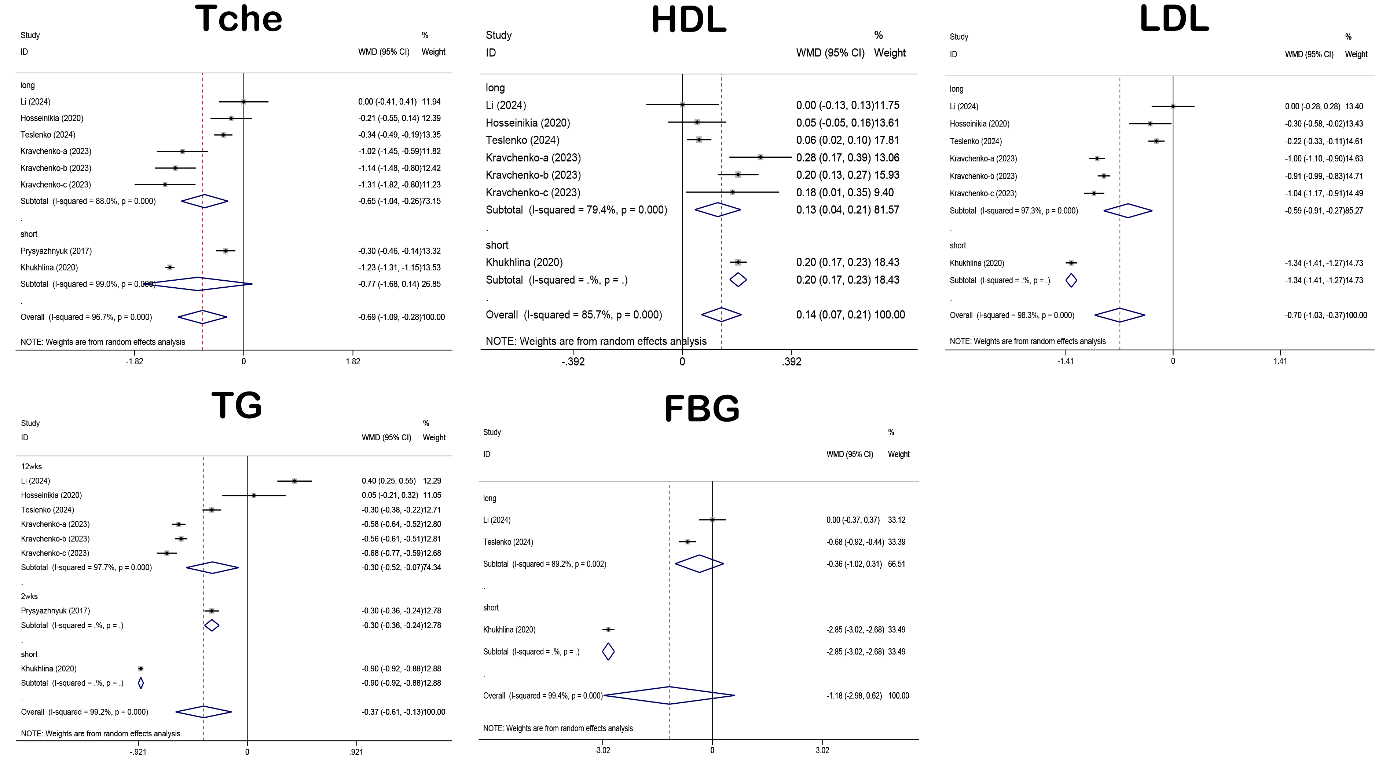


**Supplementary Figure S4** Forest plots of subgroup analyses by treatment duration on metabolic indicators. Tche: total cholesterol; HDL: high-density lipoprotein; LDL: low-density lipoprotein; TG: triglycerides.and metabolic indicators; FBG: fasting blood glucose.
